# Acidic organelles mediate TGF-β1-induced cellular fibrosis via (pro)renin receptor and vacuolar ATPase trafficking in human peritoneal mesothelial cells

**DOI:** 10.1038/s41598-018-20940-x

**Published:** 2018-02-08

**Authors:** Ikuko Oba-Yabana, Takefumi Mori, Chika Takahashi, Takuo Hirose, Yusuke Ohsaki, Satoshi Kinugasa, Yoshikazu Muroya, Emiko Sato, Geneviève Nguyen, Rémi Piedagnel, Pierre M. Ronco, Kazuhito Totsune, Sadayoshi Ito

**Affiliations:** 1 Division of Nephrology, Endocrinology and Vascular Medicine, Tohoku University Graduate School of Medicine, Sendai, Japan; 2 Division of Nephrology and Endocrinology, Tohoku Medical and Pharmaceutical University, Sendai, Japan; 3 Division of Integrated Renal Replacement Therapy, Tohoku University Graduate School of Medicine, Sendai, Japan; 4 Center for Interdisciplinary Research in Biology, Collège de France, Paris, France; 5 INSERM, UMR_S 1155, F-75020 Paris, France; 6 Sorbonne Universités, UPMC Univ Paris 06, UMR_1155, F-75005 Paris, France; 7 Department of Nephrology, AP-HP, Hôpital Tenon, Paris, France; 8 Department of Social Welfare, Faculty of Synthetic Welfare, Tohoku Fukushi University, Sendai, Japan

## Abstract

TGF-β1, which can cause renal tubular injury through a vacuolar-type H^+^-ATPase (V-ATPase)-mediated pathway, is induced by the glucose degradation product methylglyoxal to yield peritoneal injury and fibrosis. The present study investigated the roles of V-ATPase and its accessory protein, the (pro)renin receptor, in peritoneal fibrosis during peritoneal dialysis. Rats daily administered 20 mM methylglyoxal intraperitoneally developed significant peritoneal fibrosis after 7 days with increased expression of TGF-β and V-ATPase, which was reduced by the inhibition of V-ATPase with co-administration of 100 mM bafilomycin A1. The (pro)renin receptor and V-ATPase were expressed in acidic organelles and cell membranes of human peritoneal mesothelial cells. TGF-β1 upregulated the expression of collagens, α-SMA, and EDA-fibronectin, together with ERK1/2 phosphorylation, which was reduced by inhibition of V-ATPase, (pro)renin receptor, or the MAPK pathway. Fibronectin and the soluble (pro)renin receptor were excreted from cells by acidic organelle trafficking in response to TGF-β1; this excretion was also suppressed by inhibition of V-ATPase. Soluble (pro)renin receptor concentrations in effluents of patients undergoing peritoneal dialysis were associated with the dialysate-to-plasma ratio of creatinine. Together, these results demonstrate a novel fibrosis mechanism through the (pro)renin receptor and V-ATPase in the acidic organelles of peritoneal mesothelial cells.

## Introduction

Peritoneal dialysis (PD) comprises a renal replacement therapy with many unique advantages;^1^ however, peritoneal fibrosis in patients undergoing long-term PD alters peritoneal function and limits the PD treatment period^2^. Examined mechanisms of peritoneal fibrosis and alterations to peritoneal transport include inflammation^3,4^, oxidative/carbonyl stress^3–6^, the renin-angiotensin system (RAS)^5^, epithelial-to-mesenchymal transition^6^, angiogenesis^3^, and cytokines^5^, although a comprehensive understanding of these processes remains to be elucidated.

Vacuolar-type H^+^-ATPase (V-ATPase) and the (pro)renin receptor [(P)RR], an accessory protein of V-ATPase and a component of RAS, were previously demonstrated to be involved in organelle acidification and vesicle-mediated intracellular protein transport^7–9^. Together with V-ATPase, (P)RR was found to associate with cellular signaling and injury via several pathways: i) the mitogen-activated protein kinase (MAPK)-dependent pathway;^10^ ii) the Wnt/β-catenin pathway;^7^ and iii) an autophagy-mediated pathway involving V-ATPase^11^. The soluble form of (P)RR [s(P)RR] is enzymatically cleaved from full-length (P)RR [fl(P)RR] and has been detected in plasma^12^.

Transforming Growth Factor-β1 (TGF-β1) functions as a strong mediator of peritoneal fibrosis and cellular injury in human peritoneal mesothelial cells (HPMCs)^13,14^. Although TGF-β1 has been shown to be involved in renal tubular injury through SMAD3 signaling pathways^15,16^ and V-ATPase-mediated pathways^17^, little is known regarding whether this pathway is involved in the pathogenesis of peritoneal fibrosis. Moreover, methylglyoxal (MG), a glucose degradation product, has been shown to increase expression of TGF-β1 and induce peritoneal injury and fibrosis^18–20^.

Thus, we hypothesised that (P)RR and V-ATPase are expressed in HPMCs and involved in MG and TGF-β1-induced peritoneal fibrosis. To test this hypothesis, we first determined V-ATPase expression and role in the submesothelial compact zone of rats administered MG in the peritoneal cavity. Second, (P)RR and V-ATPase expression were examined in the organelles of HPMCs. Third, the functional roles of these molecules were determined in acidic organelles and cell fibrosis. Finally, the role of s(P)RR was examined in the peritoneum of patients with chronic kidney disease (CKD) undergoing PD.

## Results

### Role of V-ATPase on MG-induced peritoneal fibrosis in rats

**Figure 1:**
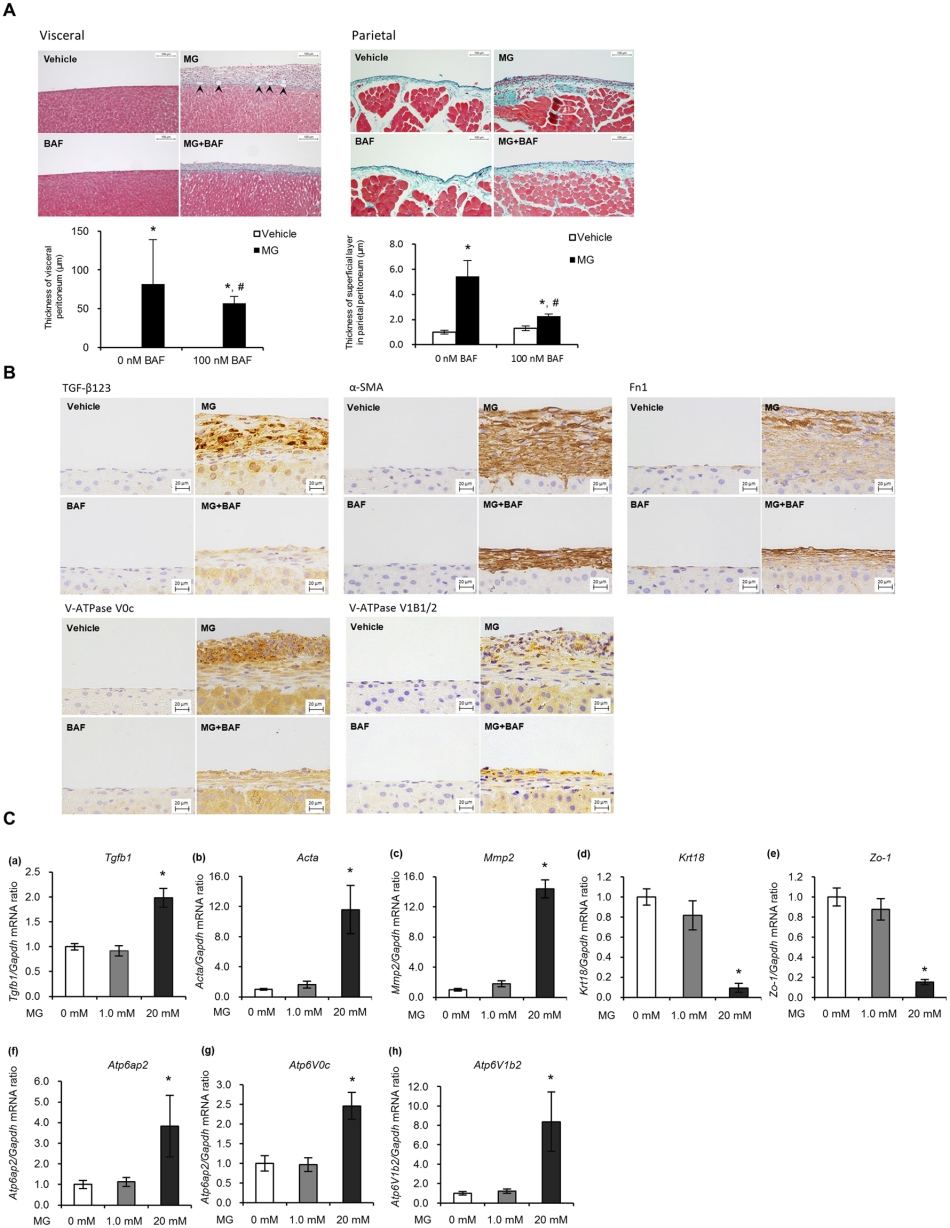
Role of V-ATPase on MG-induced peritoneal fibrosis in rats. (**A**) Representative images with Masson trichrome staining of visceral (surface of the liver) and parietal peritoneal membrane. Rats were injected intraperitoneally with standard peritoneal dialysis fluid (PDF) (Vehicle, n = 7), PDF + 20 mM MG (MG, n = 7), PDF + 100 nM BAF (BAF, n = 7), or PDF + 100 nM BAF + 20 mM MG (MG + BAF, n = 6) for 7 days. The arrow head indicates neoformed vessels induced by MG treatment.Values are expressed as the means ± SE. *P *<* 0.05 vs Vehicle. ^#^P < 0.05 vs 0 nM BAF. Scale bars, 100 µm. (**B**) Immunohistochemical staining of TGF-β123, α-SMA, Fn1, Atp6v0c, and Atp6v1b1/2 in the visceral peritoneum of 4 groups. Scale bars, 20 µm. (**C**) mRNA expression of TGF-β1, fibrosis markers (Acta, Mmp2), epithelial markers (Krt18, Zo1) and V-ATPase subunits (Atp6ap2/(P)RR, Atp6v0c, Atp6v1b2) in rat peritoneal mesothelial cells. Rats were treated with single peritoneal injection of PDF containing 0, 1.0 or 20 mM MG (n = 4–7). Values are expressed as the means ± SE. *P < 0.05 vs 0 mM MG.

To determine whether V-ATPase is involved in peritoneal fibrosis, we first utilised the MG-induced peritoneal fibrosis model in Wistar rats. MG induced significant thickening of the fibrotic submesothelial compact zone in the visceral peritoneal membrane (Fig. 1A). Similarly, this thickening was also observed in the parietal membrane (Fig. 1A). Intraperitoneal administration of the V-ATPase inhibitor bafilomycin A1 (BAF) significantly attenuated MG-induced thickening, indicating that V-ATPase was responsible for the MG-mediated peritoneal fibrosis. Broad expression of TGF-β123, α-SMA (Acta), Fibronectin (Fn1), Atp6v0c, and Atp6v1b1/2 was observed in the visceral peritoneal membrane fibrotic submesothelial compact zone, which was also inhibited by BAF (Fig. 1B). It is difficult to assess V-ATPase expression by western blotting and real-time quantitative PCR (qPCR), because we cannot specifically pick the thickening area of the peritoneum, especially in Vehicle or BAF group (Fig. 1A). Thus, we used human peritoneal mesothelial cells in the following experiments.

Next, to determine whether TGF-β1 and V-ATPase/(P)RR are involved in MG-induced fibrosis, we determined the role of V-ATPase and (P)RR in rat peritoneal mesothelial cells (RPMCs). The mRNA expression of *Tgfb1*, (P)RR (*Atp6ap2*), V-ATPase subunits (*Atp6v0c*, *Atp6v1b2*) and fibrosis markers (*Acta*, *Mmp2*) was significantly increased after intraperitoneal administration of 20 mM MG for 3 days (Fig. 1C). Conversely, mRNA expression of epithelial markers (*Krt18*, *Zo-1*) was significantly reduced, indicating RPMCs transformation (Fig. 1C).

### Localisation of (P)RR and V-ATPase in the organelles of HPMCs

**Figure 2:**
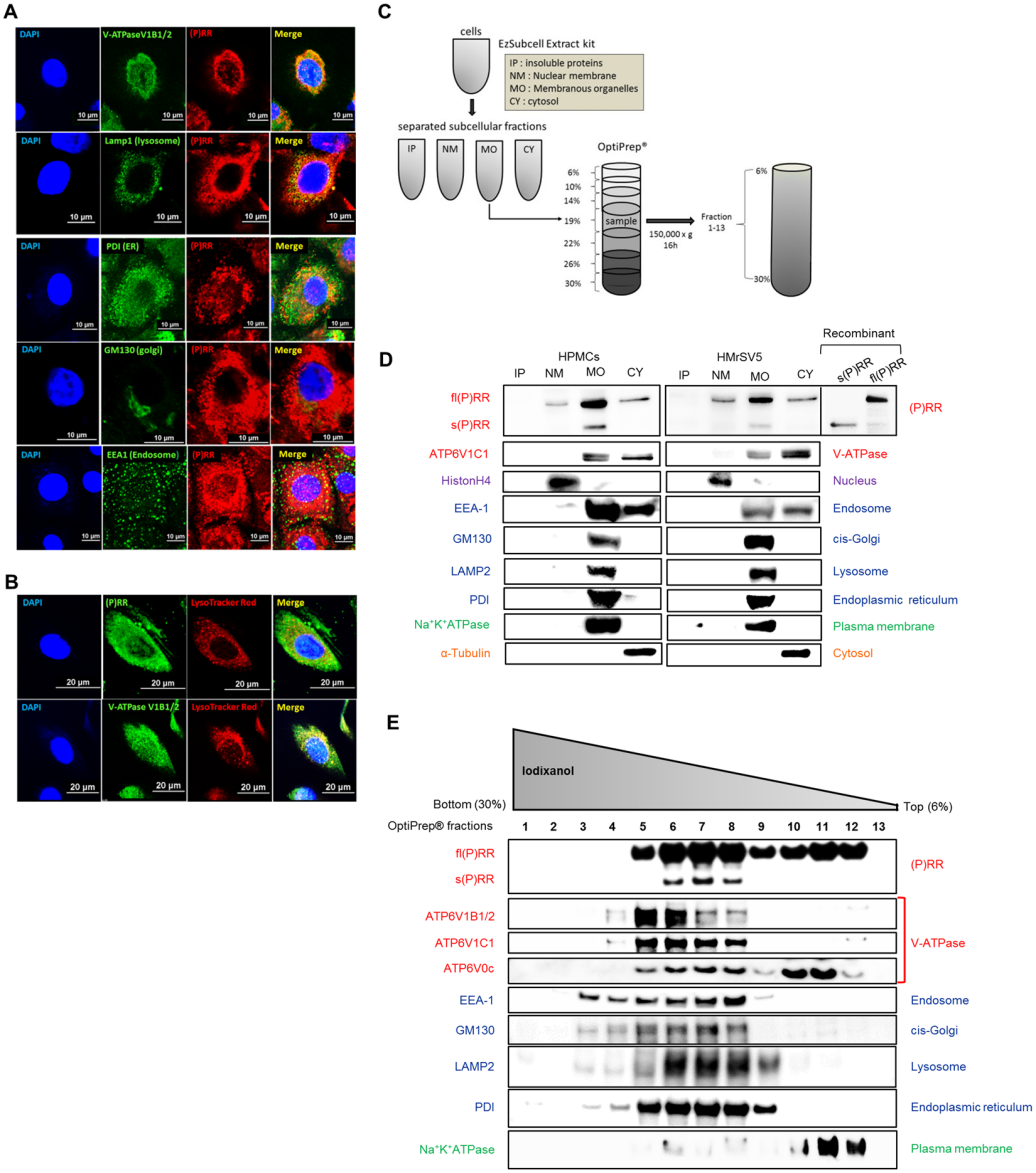
Expression of (P)RR and V-ATPase in the organelles of primary human peritoneal mesothelial cells (HPMCs). (**A**) Intracellular co-localisation of (P)RR (red) and acidic organelles (green) in HPMCs. HPMCs were immunohistochemically co-stained with (P)RR, and V-ATPase subunit (ATP6V1B1/2), LAMP1, PDI, GM130, or EEA-1. Scale bars, 10 µm. (**B**) Co-localisation of (P)RR and V-ATPase subunit (ATP6V1B1/2) with acidic organelles in HPMCs. Acidic organelles were labelled with LysoTracker Red (red) and (P)RR or ATP6V1B1/2 (green) was stained immunohistochemically. Nuclei were stained with DAPI (4′,6-diamidino-2-phenylindole, blue). Scale bars, 20 µm. (**C**) Schematic image of the organelles isolation. Subcellular fractions of HPMCs were isolated using the EzSubcell extract kit. The membranous organelle fraction was applied to an OptiPrep gradient (6–30%) and ultracentrifuged at 150,000 × *g* for 16 hours. Thirteen fractions were collected bottom to top from the OptiPrep gradient. (**D**) Protein expression in subcellular fractions of HPMCs and HMrSV5 cells. The cells were separated into the insoluble protein (IP), nuclear membrane (NM), membranous organelle (MO), and cytosol (Cy) by EzSubcell Extract kit. Western blotting analysis of each crude subcellular fraction was performed for (P)RR and ATP6V1C1, nuclear (Histone H4), organelle (EEA-1, GM130, LAMP2, PDI), plasma membrane (Na^+^K^+^ATPase), and cytosolic (α-tubulin) marker proteins. Recombinant full-length [fl(P)RR] and soluble (P)RR [s(P)RR] are shown as positive controls. (**E**) Expression of (P)RR and V-ATPase subunits (ATP6V1B1/2, ATP6V1C1, ATP6V0c) in the 13 membranous organelle fractions by OptiPrep density gradient centrifugation.

(P)RR was expressed in HPMCs (Supplementary Fig. S1). To clarify the subcellular localisation of (P)RR and V-ATPase (ATP6V1B1/2) subcellular localisation, we performed immunofluorescence. (P)RR and ATP6V1B1/2 co-localised in primary HPMCs (Fig. 2A), compatible with previous reports that (P)RR constitutes a V-ATPase accessory protein^12,21^. Double staining immunofluorescence revealed that (P)RR localised with the lysosome (LAMP1), and also with endoplasmic reticulum (ER) (PDI), Golgi apparatus (GM130), and endosome (EEA1), suggesting that (P)RR and V-ATPase are expressed in these organelles, albeit at lower levels than those of lysosomes (Fig. 2A). To determine whether (P)RR is responsible for HPMC organelle acidification, cells were loaded with the fluorescent acidic organelle sensor LysoTracker Red followed by immunofluorescence with (P)RR or ATP6V1B1/2. LysoTracker Red co-localised with (P)RR or ATP6V1B1/2 (Fig. 2B).

To confirm (P)RR and V-ATPase expression in acidic organelles, we isolated subcellular fractions from HPMCs and HMrSV5 (Fig. 2C). We first used an EzSubcell Extract kit to obtain four crude subcellular fractions containing different membrane markers (cytosol, membranous organelle, nuclear membrane, and insoluble protein). The nuclear membrane fraction mainly expressed HistonH4, a nuclear marker. The membranous organelle fraction expressed LAMP2, EEA-1, GM130, PDI, and Na^+^K^+^ ATPase, which constitute lysosomal, endosomal, Golgi apparatus, ER, and plasma membrane markers, respectively (Fig. 2D). As fl(P)RR and s(P)RR were primarily observed in the membranous organelle fraction, which also contained ATP6V1C1 in both HPMCs and HMrSV5 cells, we further prepared 13 fractions from the HPMC membranous organelle fraction by OptiPrep gradient density centrifugation (Fig. 2C). fl(P)RR was distributed broadly through fractions 5–12, which was present in the lysosome and plasma membrane fractions. fl(P)RR also partially localized in the ER, the endosome, and the Golgi apparatus. Conversely, s(P)RR was distributed in fractions 6–8, which present in the membranous organelle fraction. Furthermore, the V-ATPase V1 domain (ATP6V1B1/2 and ATP6V1C1) was mainly distributed in most of organelle fractions (fractions 4–8), whereas the V0 domain (ATP6V0c) was distributed in fractions 5–13, similar to that of fl(P)RR (Fig. 2E). These results indicated that (P)RR and V-ATPase were widely distributed in lysosomes, some elements of the ER, the endosomes, and the cis-Golgi, all of which comprise acidic organelles. The s(P)RR and V-ATPase V1 domains were exclusively distributed only in organelles, whereas the fl(P)RR and V-ATPase V0 domains were distributed in both organelles and the plasma membrane.

### Role of (P)RR and V-ATPase in TGF-β1-induced HPMC fibrosis

**Figure 3:**
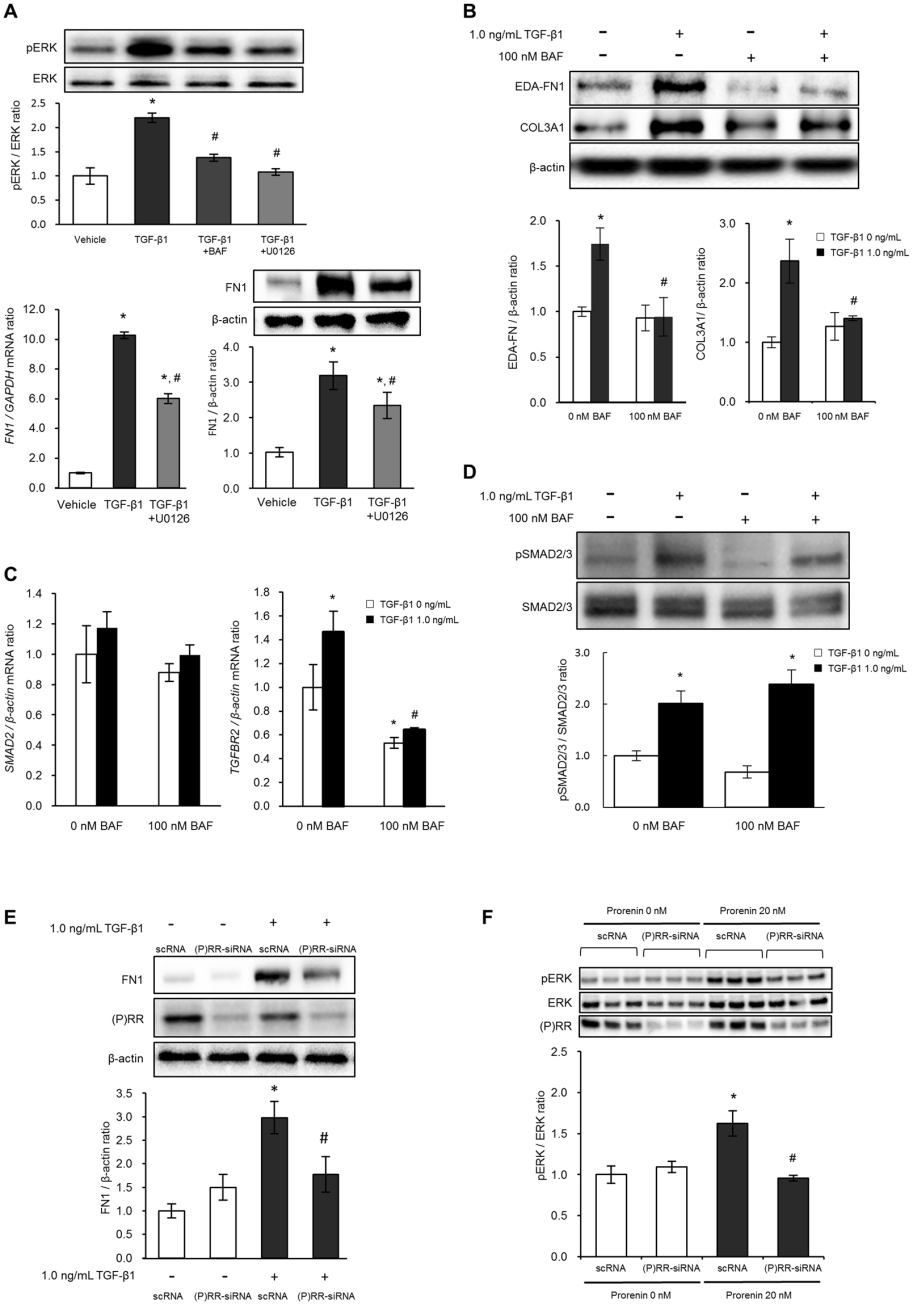
Role of V-ATPase and MAPK pathway in TGF-β1-induced fibrosis in peritoneal mesothelial cells. (**A**) TGF-β1-mediated ERK1/2 phosphorylation, and the effect of 100 nM BAF (V-ATPase inhibitor) or 10 μM U0126 (MEK1/2 inhibitor) in HMrSV5 cells. Values are expressed as the means ± SE (n = 4). *P < 0.05 vs vehicle, ^#^P < 0.05 vs TGF-β1. (**B**) Representative images and quantitative analysis of EDA-FN1 and COL3A1 in HMrSV5 cells. Values are expressed as the means ± SE (n = 3). *P < 0.05 vs TGF-β1 (−)/BAF (−), ^#^P < 0.05 vs TGF-β1 (+)/BAF (−). (**C**) The effect of V-ATPase inhibition for expression of SMAD2 and TGFBR2 on TGF-β1 induced fibrosis in peritoneal mesothelial cell. HMrSV5 cells were pretreated with 0 or 100 nM BAF for 1 hour, and then treated with 0 or 1.0 ng/mL of TGF-β1 for 24 hours. Values are expressed as means ± SE (n = 3). *P < 0.05 vs TGF-β1 (−)/BAF (−), ^#^P < 0.05 vs TGF-β1 (−)/BAF (+). (**D**) The effect of V-ATPase inhibition for SMAD2/3 phosphorylation on TGF-β1 induced fibrosis in peritoneal mesothelial cell. HMrSV5 cells were pretreated with 0 or 100 nM BAF for 1 hour, and then treated with 0 or 1.0 ng/mL of TGF-β1 for 24 hours. Values are expressed as means ± SE (n = 6). *P < 0.05 vs TGF-β1 (−)/BAF (−), ^#^P < 0.05 vs TGF-β1 (−)/BAF (+). (**E**) Representative image and quantitative analysis of FN1 in HMrSV5 cells. Treatment: scRNA or (P)RR-siRNA for 48 hours stimulation of 1.0 ng/mL TGF-β1. Values are expressed as the means ± SE (n = 6). *P < 0.05 vs TGF-β1 (−), ^#^P < 0.05 vs scRNA. (**F**) Effect of (P)RR knockdown on prorenin-stimulated ERK1/2 phosphorylation in HMrSV5 cells. Treatment: scRNA or (P)RR-siRNA for 48 hours for 5 min stimulation of 20 nM prorenin (n = 6). Values are expressed as the means ± SE (n = 6). *P < 0.05 vs 0 nM prorenin, ^#^P < 0.05 vs 20 nM prorenin treatment.

TGF-β1 dose-dependently stimulated FN1 expression, and 100 nM BAF reduced this response and mRNA expression of fibrosis markers (Supplementary Fig. S2). However, TGF-β1 did not directly increase (P)RR (*ATP6AP2*) mRNA (n = 4, P = 0.551, Supplementary Fig. S3A). We then determined the role of the MAPK pathway in TGF-β1-induced fibrosis. TGF-β1 at 1.0 ng/mL significantly induced pERK at 24 hours, which was inhibited by BAF and the MEK1/2 inhibitor U0126 (Fig. 3A), at a dose consistent with that of a prior report^22^. U0126 (10 µM) significantly but partially attenuated FN1 mRNA and protein response to TGF-β1 (P* < *0.05) and inhibited TGF-β1-induced cellular FN1 expression. These results indicate that the MAPK pathway is responsible for TGF-β1-induced fibrosis in HPMCs.

Evidence suggests that fibroblast differentiation into myofibroblasts requires the combined action of mechanical tension, TGF-β1, and the FN1 splicing variant containing extra domain A (EDA-FN1)^23^. The association between V-ATPase and TGF-β1-induced fibrosis was also confirmed by western blotting, which demonstrated that EDA-FN1 and COL3A1 expression in response to 1.0 ng/mL TGF-β1 was reduced by 100 nM BAF treatment (Fig. 3B). To confirm whether V-ATPase is effect on the TGF-β1 mediated SMAD pathway, the expression of SMAD2 and TGFBR2 mRNA (Fig. 3C) and the phosphorylation of SMAD2/3 (Fig. 3D) were evaluated. Stimulation with 1.0 ng/mL TGF-β1 for 24 hours increased SMAD2/3 phosphorylation, but no effects on SMAD2 mRNA expression was observed. Treatment with 100 nM BAF did not alter both SMAD2 mRNA expression and SMAD2/3 phosphorylation. In contrast, 1.0 ng/mL of TGF-β1 significantly increased TGFBR2, which was inhibited by 100 nM BAF treatment. As V-ATPase inhibition may alter cell viability, we determined whether inhibition of (P)RR, a V-ATPase accessory protein, could reduce TGF-β1-mediated FN1 expression. (P)RR-short interfering RNA (siRNA) knockdown of (P)RR significantly reduced the FN1 increase induced by 1.0 ng/mL TGF-β1 stimulation for 24 hours (Fig. 3E). We also determined whether direct (P)RR stimulation by prorenin might induce ERK1/2 phosphorylation. In the presence of scRNA, 20 nM prorenin increased the pERK/ERK ratio, which was inhibited by (P)RR-siRNA (Fig. 3F, Supplementary Fig. S4). In addition, the 20 nM of prorenin failed to induce TGF-β1 (TGFB1) mRNA in HMrSV5 cells with/without losartan and PD123319, and there were no dose-dependent increases in TGFB1 (n = 3, P = 0.550) mRNA up to 200 nM of prorenin (Supplementary Fig. S5).

### Role of acidic organelles in TGF-β1-induced FN1 accumulation and fibrosis

**Figure 4:**
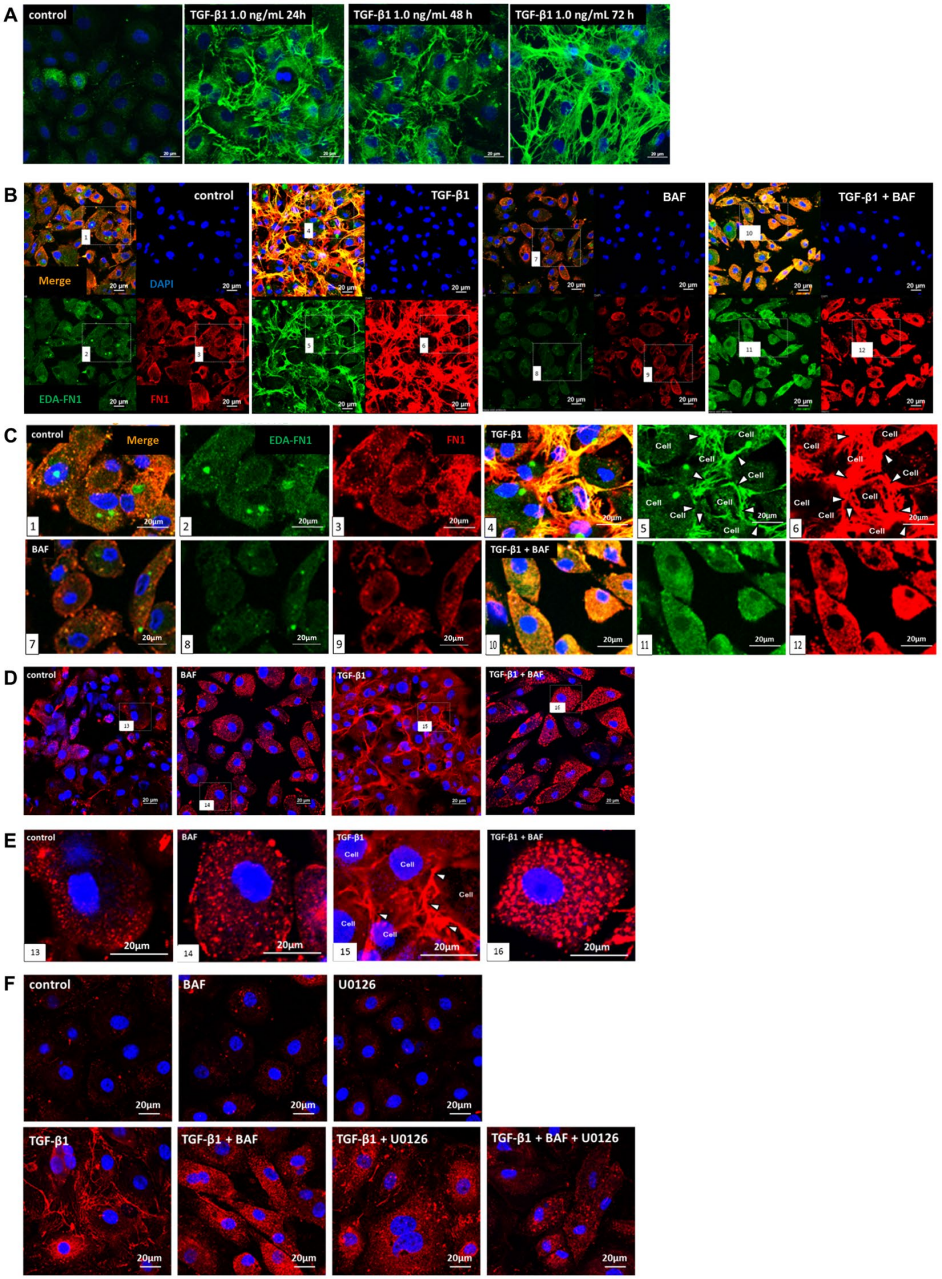
Role of V-ATPase in the TGF-β1-induced fibrosis of human peritoneal mesothelial cells (HPMCs). (**A**) Time course of FN1 (green) accumulation in response to TGF-β1 stimulation in HPMCs. Cells were pretreated with or without 1.0 ng/mL TGF-β1 for 24, 48, or 72 hours. Nuclei were stained with DAPI (4′,6-diamidino-2-phenylindole, blue). Scale bars, 20 µm. (**B**) Localisation of profibrotic molecules [EDA-FN1 (green) and FN1 (red)] in response to TGF-β1 stimulation and V-ATPase inhibition (BAF) in HPMCs. Cells were pretreated with or without 100 nM BAF for 1 hour prior to 0 or 1.0 ng/mL TGF-β1 treatment for 24 hours. Nuclei were stained with DAPI (blue). Scale bars, 20 µm. (**C**) Enlarged views of the white box areas in 1–12. Arrowheads indicate EDA-FN1 or FN1 accumulation between cells. Scale bars, 20 µm. (**D**) Effect of TGF-β1 and V-ATPase inhibition (BAF) on intracellular collagen IV (red) localisation in HPMCs. Cells were pretreated with or without 100 nM BAF for 1 hour prior to 0 or 1.0 ng/mL TGF-β1 for 24 hours. Nuclei were stained with DAPI (blue). Scale bars, 20 µm. (**E**) Enlarged views of the white box areas in 13–16. Arrowheads indicate the collagen IV accumulation between cells. Scale bars, 20 µm. (**F**) TGF-β1-induced FN1 cell accumulation was inhibited by the MEK inhibitor U0126, which was similar to that of BAF. Scale bars, 20 µm.

FN1 and EDA-FN1 accumulation was determined by immunofluorescence. Cell surface FN1 accumulation in response 1.0 ng/mL TGF-β1 treatment for 24, 48, or 72 hours was observed in HPMCs (Fig. 4A). This response was attenuated by 100 nM BAF. However, as compared with BAF alone, high FN1 and EDA-FN1 levels in the vesicles were observed (Fig. 4B,C). To determine whether FN1 and EDA-FN1 accumulation contributes to fibrosis, the collagen IV response to TGF-β1 was also determined. Both FN1 and EDA-FN1 accumulated at the cell surface in response to 1.0 ng/mL TGF-β1. This response was attenuated by the presence of BAF, although the intracellular FN1 or EDA-FN1 vesicular accumulation remained. Collagen IV demonstrated a similar pattern as FN1 or EDA-FN1, both in response to TGF-β1 and following BAF treatment (Fig. 4D,E). U0126 inhibited cell surface accumulation of FN1, which remained in the vesicles following BAF treatment (Fig. 4F).

### Excretion of soluble (P)RR and the fibrotic matrix by HPMC acidic organelles

**Figure 5:**
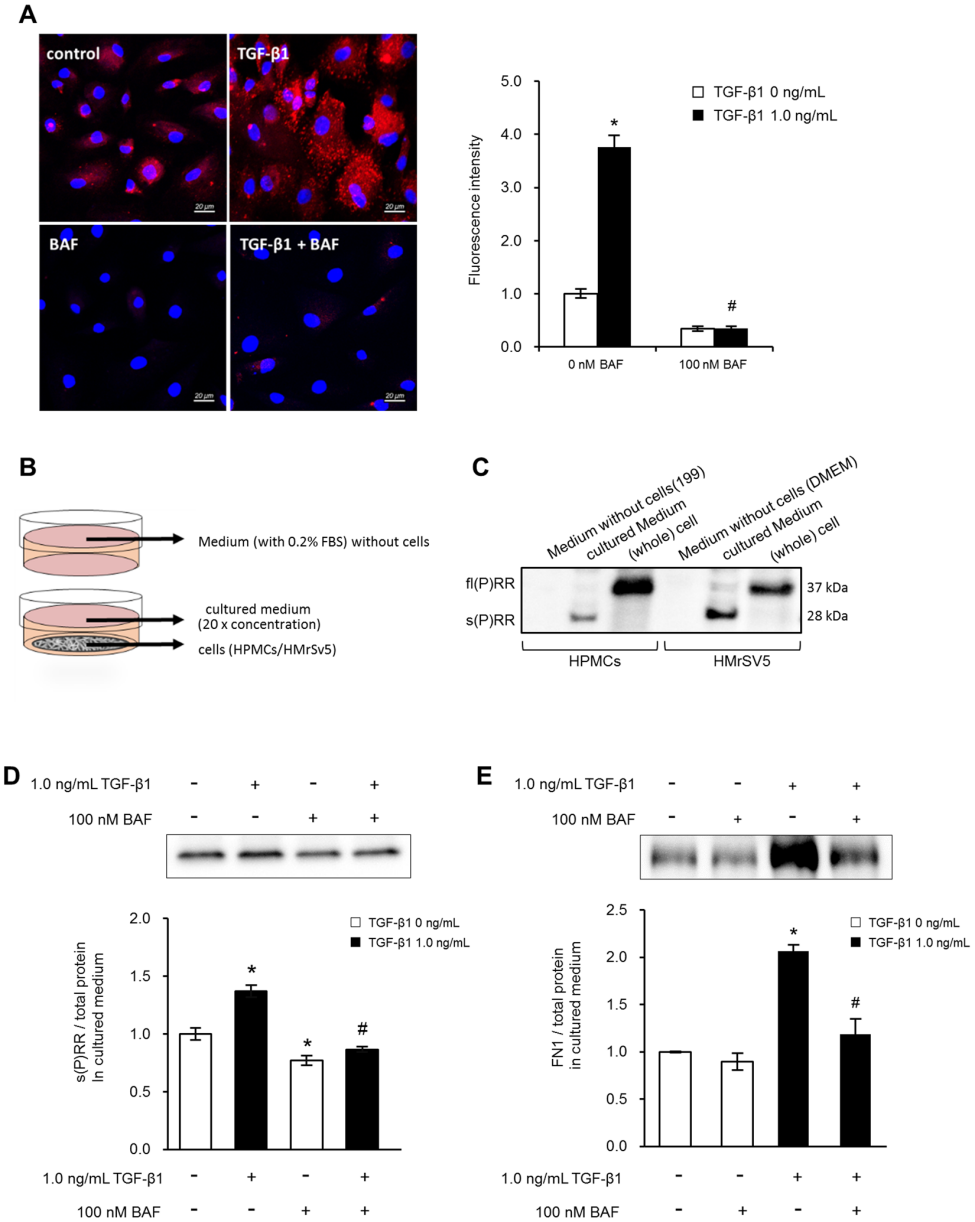
Role of V-ATPase activity and cellular trafficking of profibrotic molecules in the TGF-β1-induced fibrosis of human peritoneal mesothelial cells (HPMCs). (**A**) Role of TGF-tracking of profibrotic molecules in the TGF-β1-induced fibrosis in HPMCs. Cells were pretreated with or without 100 nM BAF for 1 hour, and treated with 1.0 ng/mL TGF-β1 for 24 hours. Acidic organelle activity was determined with LysoTracker Red. Values are the average positive areas from individual cells in six separate dishes, and expressed as the means ± SE (n = 6). *P < 0.05 vs 0 ng/mL TGF-β1, ^#^P < 0.05 vs 0 nM BAF. Scale bars, 20 µm. (**B**) Schematic illustration of the experimental design for cellular trafficking by V-ATPase inhibition on s(P)RR secretion in primary HPMCs and cultured HPMCs (HMrSV5 cells). (**C**) Representative blotting of concentrated (20× ) medium with and without cells and whole cells. (**D**) s(P)RR expression in the conditional medium. HMrSV5 cells were pretreated with 0 or 100 nM BAF for 1 hour, and treated with 1.0 ng/mL TGF-β1 for 24 hours. Values are expressed as the means ± SE (n = 3). *P < 0.05 vs 0 ng/mL TGF-β1, ^#^P < 0.05 vs 0 nM BAF. (**E**) FN1 expression in the conditional medium. HMrSV5 cells were pretreated with 0 or 100 nM BAF for 1 hour, and treated with 1.0 ng/mL TGF-β1 for 24 hours. Values are expressed as the means ± SE (n = 6). *P < 0.05 vs 0 ng/mL TGF-β1; ^#^P < 0.05 vs 0 nM BAF.

The role of V-ATPase in acidic organelles was determined by 100 nM BAF treatment in the presence or absence of 1.0 ng/mL TGF-β1. TGF-β1 increased the LysoTracker Red signal, which was abolished by 100 nM BAF (Fig. 5A). We next investigated fl(P)RR and s(P)RR cellular expression and extracellular excretion as shown in Fig. 5B. fl(P)RR expression was observed in whole cells; conversely, s(P)RR expression was identified in the medium in which HPMCs or HMrSV5 cells were cultured for 24 hours, after 20× concentration (Fig. 5C).

To determine the role of V-ATPase and acidic organelles in s(P)RR and matrix trafficking, s(P)RR and FN1 levels in response to 100 nM BAF treatment were determined.

TGF-β1 stimulated the s(P)RR and FN1 extracellular excretion to the culture medium and which was inhibited by 100 nM BAF treatment (Fig. 5D,E). Taken together, these results indicate that both s(P)RR and FN1 are secreted from the cell through trafficking by acidic organelles.

### Role of soluble (P)RR in PD fluid

**Figure 6:**
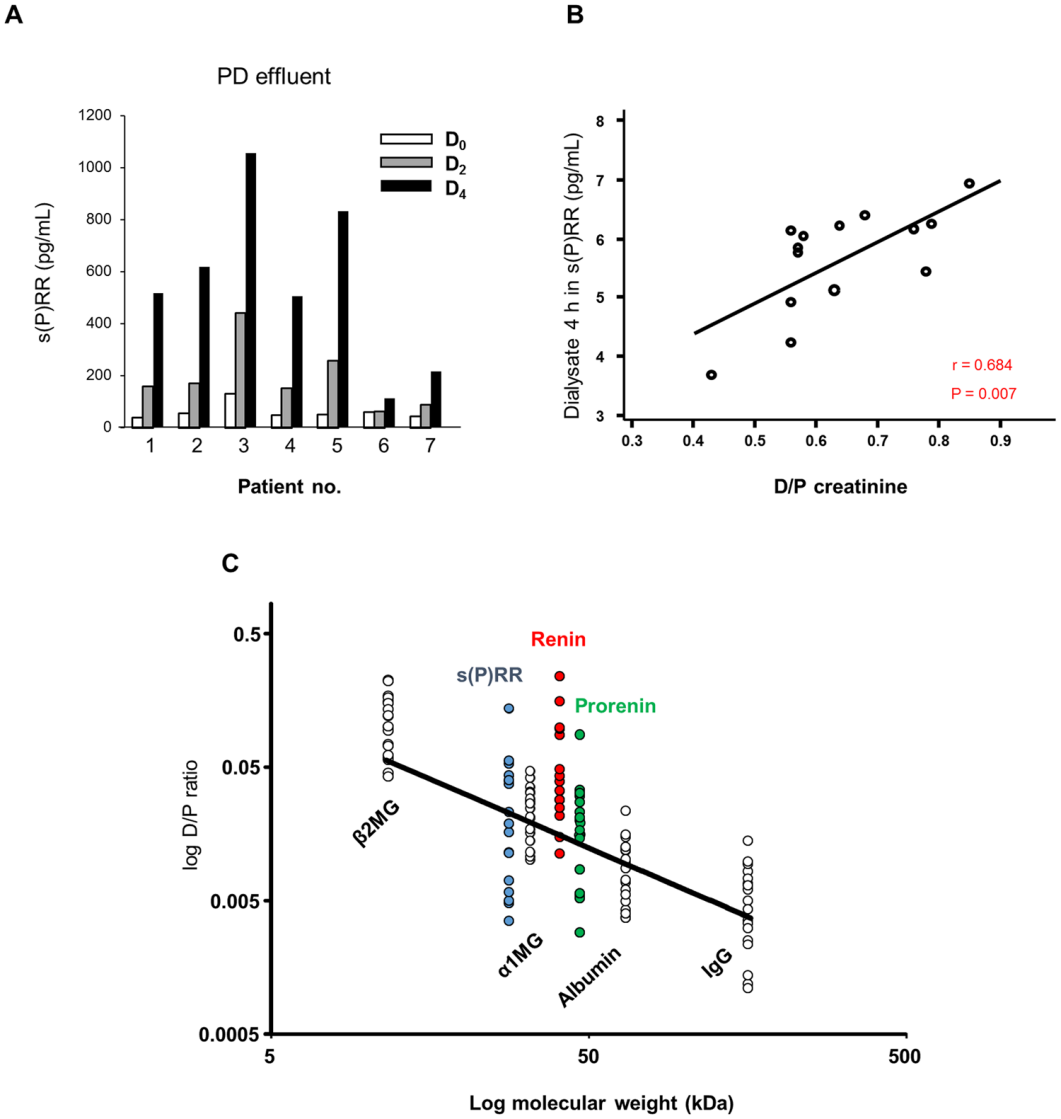
s(P)RR concentrations in patients with CKD on regular peritoneal dialysis treatment. (**A**) s(P)RR concentrations in PDEs obtained at 0, 2, and 4 hours during peritoneal equilibration tests (D0, D2, D4, respectively, n = 7). (**B**) Correlation between the dialysate-to-plasma (D/P) creatinine ratio (D/P Cr) and s(P)RR in PDEs at 4 hours (r = 0.684). (**C**) Relationship between molecular weight and log D/P of β2 microglobulin (β2MG, 11.8 kDa), α1 microglobulin (α1MG, 33 kDa), albumin (66 kDa), immunoglobulin G (IgG, 160 kDa), s(P)RR (28 kDa), renin (40 kDa), and prorenin (46 kDa) ratio.

As s(P)RR was observed to be secreted from HPMCs, s(P)RR levels in the plasma and peritoneal dialysate effluents (PDEs) from patients with CKD on regular PD treatment (men = 5, women = 2; origin of renal failure: diabetic nephropathy, n = 2; hypertensive nephrosclerosis, n = 2; chronic glomerulonephritis or unknown, n = 3, no previous episodes of peritonitis) were analysed. Administration of 2.5% glucose dialysate dose-dependently increased s(P)RR concentration after 2 and 4 hours (Fig. 6A), and was associated with the dialysate-to-plasma (D/P) creatinine ratio (D/P Cr, r = 0.684, P *< *0.05; Fig. 6B). The D/P ratios of s(P)RR, renin, and prorenin did not correlate well with their molecular weight (Fig. 6C). As previously suggested^24^, these results indicated that s(P)RR concentration is determined, at least in part, by factors other than molecular weight, such as local secretion by HPMCs.

## Discussion

The current study demonstrated that MG increases TGF-β1 and V-ATPase expression in the peritoneal submesothelial compact zone and that this pathway is responsible for peritoneal fibrosis in rats. In addition, (P)RR and V-ATPase in HPMC acidic organelles play a role in profibrotic molecule cellular signaling and trafficking as reported previously in other cell types^8,25^. To our knowledge, this is the first study that demonstrated the role of V-ATPase to the secretion of s(P)RR and fibrosis in HPMCs. Furthermore, s(P)RR levels in PD fluid, which is excreted from mesothelial cells, correlated with the peritoneal injury. This V-ATPase- and (P)RR-mediated acidic organelle trafficking of profibrotic molecules in peritoneal mesothelial cells represents a novel finding and provides new insights into the mechanisms of peritoneal fibrosis.

Initially, we determined whether V-ATPase is responsible for MG-induced peritoneal fibrosis. MG-induced peritoneal injury is well documented by Hirahara *et al*.^26^, who additionally reported that MG upregulated TGF-β1 and collagen 1 expression in rat peritoneum. However, inducing fibrosis through simply administering TGF-β1 into the rat peritoneal cavity is difficult. Some previous studies used adenovirus to induce TGF-β1 expression in the peritoneum^14^, although this technique is not available in our laboratory. Therefore, we utilised the MG-induced peritoneal fibrosis model in our study and demonstrated that MG induces peritoneal submesothelial compact zone thickening. In accordance with the HPMC experiment results, V-ATPase was increased in the submesothelial compact zone, together with TGF-β1 and α-SMA expression. These responses were inhibited by BAF, indicating that peritoneal fibrosis in rodents is mediated by V-ATPase. Notably, baseline expression of V-ATPase and (P)RR was consistent with previous studies and co-localised in acidic organelles^21,27^. The comparative analysis of visceral and parietal peritoneum for peritoneal membrane function is mostly unknown. In the present study, the characteristics of visceral and parietal peritoneum are different as previously reported^28^. In case of encapsulating peritoneal sclerosis (EPS), a feature is almost exclusively in the visceral peritoneal membrane^29^. Therefore we have focused on visceral peritoneum.

TGF-β1 is considered the master molecule in the genesis of peritoneal fibrosis, and is widely used for cell fibrosis experiments in HPMCs and *in vivo*^13–16,30,31^. TGF-β1 induced FN1 expression dose-dependently in the present study. As MAPK has been shown to play a role in fibronectin expression in HPMCs^32^, we assessed the MAPK pathway in our model. V-ATPase, MEK1/2 inhibition and (P)RR knockdown inhibited TGF-β1 induced fibrosis in HPMCs. BAF inhibited TGF-β1-induced ERK1/2 and U0126 inhibited FN1 trafficking. These results suggest that V-ATPase and (P)RR are responsible for TGF-β1-induced FN1 expression via the MAPK pathway. In addition, it has been demonstrated that Smad3 may be the most prominent downstream mediator in TGF-β-induced peritoneal fibrosis^15,16^. We also focused on the SMAD signaling pathway. The phosphorylation of SMAD2/3 is upregulated without an increase of SMAD2 expression and BAF failed to inhibit this phosphorylation. On the other hand, the mRNA expression of TGFBR2 was significantly increased by TGF-β1 stimulation, which was inhibited by BAF. Therefore V-ATPase inhibition may have a small effect on Smad2/3 signaling and block cell surface accumulation of the profibrotic molecules after the transcription.

V-ATPase and (P)RR have been shown to be expressed in the acidic organelles of various cells and play a different pathophysiological role in each cell type, such as renal acidification, bone metabolism, and insulin secretion^33^. We confirmed whether acidic organelles are responsible for the TGF-β1-induced FN1 cell surface accumulation in HPMC. This cell fibrosis was abolished by BAF, inhibition of V-ATPase. Notably, FN1 accumulated in cellular vesicles. Thus, we conducted an experiment using LysoTracker Red, an indicator of acidic organelle activity. TGF-β1 increased the LysoTracker Red signal in the HPMC vesicles, whereas BAF greatly reduced this signal. These results indicate that TGF-β1 increases V-ATPase activity and may alter intracellular/organelle pH without an increase in V-ATPase expression. In addition, BAF inhibited s(P)RR and FN1 secretion into the medium, suggesting that molecules related to fibrosis are also secreted out of the cells by acidic organelles in HPMCs. Taken together, the results in the present study explain the novel role of acidic organelles in TGF-β1-induced HPMC fibrosis, mediated by V-ATPase and (P)RR.

Furthermore, as shown in Fig. 6, s(P)RR was detected in the PDEs of all patients in a time-dependent manner, its levels in the PDEs were significantly correlated with D/P Cr, and D/P s(P)RR concentration was on the liner regression equation of the molecular weight like prorenin and renin. Previous report has suggested that concentration of the molecules in the dialysate correlates with the size of the molecule. If the concentration is not fit with the estimated concentration, it is considered to the local excretion^24^. Although it is possible that the charge of this protein may have affected the disassociation with molecular weight, it has been demonstrated that albumin diffusion was not affected by its charge^34^. Our results indicate that s(P)RR in the dialysate is, at least in part, due to local secretion from HPMCs. However, we cannot fully conclude with this single data that s(P)RR is excreted out from peritoneum. Thus, we measured s(P)RR in the medium of cultured HPMCs, and found that s(P)RR and FN1 is excreted out of the cell. Taken together, we concluded that s(P)RR is secreted out of the cell and could be an indicator of peritoneal injury.

Although the D/P Cr ratio in itself cannot be considered as a fully reliable marker of peritoneal injury, an increase in the D/P Cr ratio is considered to be associated at least in part with peritoneal injury^35^. PDEs were collected from basically the same patients with CKD, without prior peritonitis. We excluded patients with prior peritonitis, as increased s(P)RR owing to inflammation may be present, making it difficult to evaluate V-ATPase activity or peritoneal injury. The relationship between peritoneal tissue histology and s(P)RR levels in PDEs therefore requires further study.

The present study has several limitations. Peritoneal fibrosis occurs not only in HPMCs, and interactions between other cells such as endothelial cells, pericytes, and fibroblasts may exist. It must also be acknowledged that BAF demonstrates cell toxicity at higher doses and longer durations of exposure *in vitro*; thus, we used BAF at doses and exposure durations that do not reduce cell viability^36^. However, cell number was decreased after 48 hours of BAF exposure at a dose of 100 nM (data not shown), we used 100 nM BAF for 24 hours *in vitro*. Additionally, BAF might not be an ideal drug to demonstrate *in vivo* effects because of cell toxicity with higher concentration. Although there is no information available in the previous studies, the concentration that was not toxic to the cell culture was injected in our *in vivo* experiment. We used this drug to determine the effect of total V-ATPase. To demonstrate the *in vivo* effects of specific V-ATPase or (P)RR, there are methods using V-ATPase/(P)RR vector with adenovirus or using V-ATPase/(P)RR KO mice. However, these models with selectively inhibit V-ATPase/(P)RR in the peritoneum are not yet to be established, which would be a future study. Therefore, we used (P)RR siRNA in HPMCs to selectively inhibit (P)RR. As V-ATPase plays a fundamental role in cell viability, we inhibited its accessory protein, (P)RR, by (P)RR-siRNA. HPMCs isolated from PDEs represent cells that have detached from the peritoneal membrane, which may have different characteristics from those of HPMCs in the intact peritoneum. To address this issue, we also used HMrSV5 cells, which were originally isolated from the omentum of patients without CKD.

**Figure 7:**
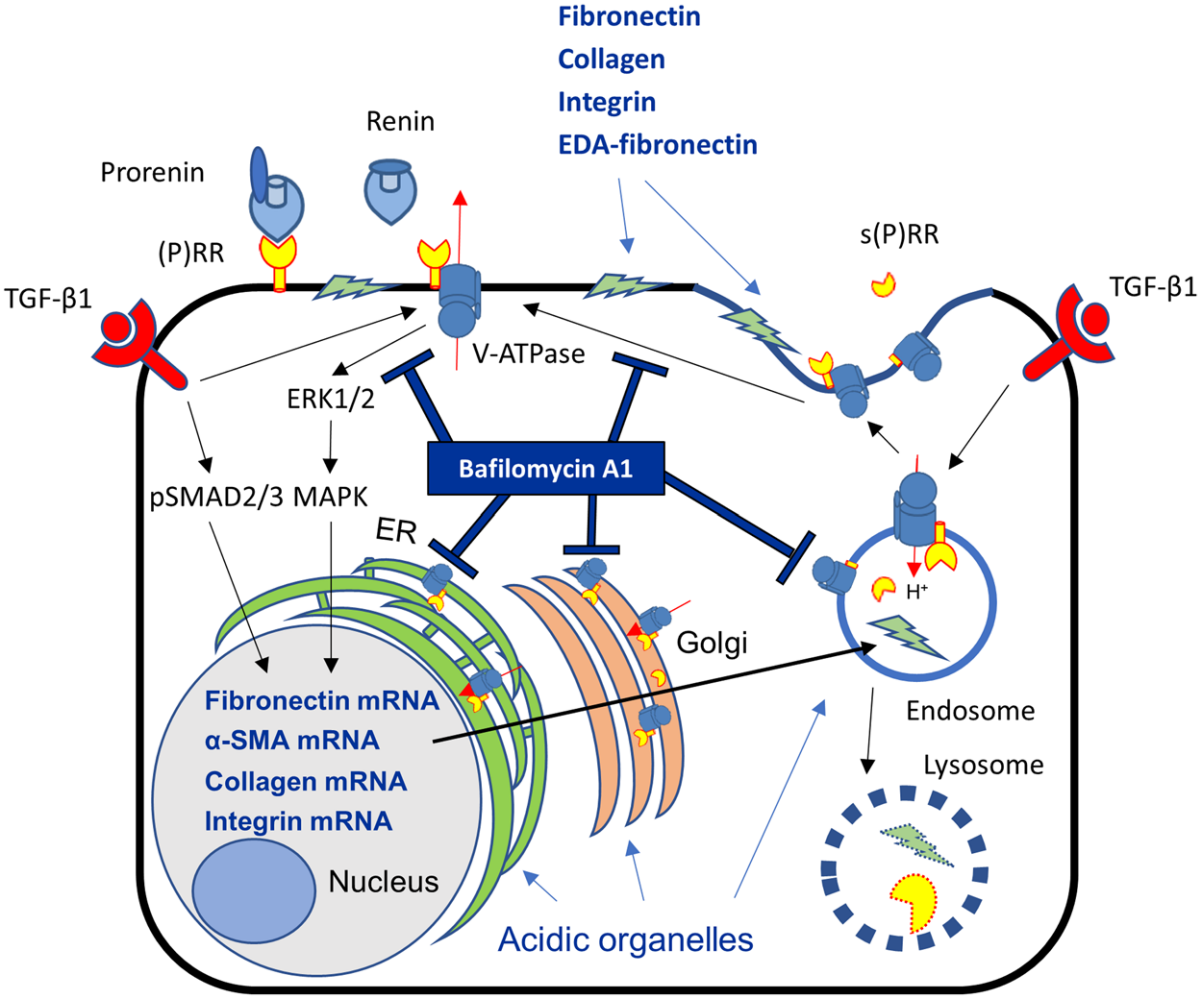
Proposed mechanism of cellular fibrosis regulated by acidic organelles. Full-length (P)RR and V-ATPase are expressed in the membranes of acidic organelles, and play a functional role in cell trafficking. TGF-β1-induced FN1 is delivered by acidic organelles to the cell membrane inside vesicles and participates in cell adhesion and fibrosis. Inhibition of V-ATPase or (P)RR inhibits cell trafficking of FN1, thereby inhibiting fibrosis. (P)RR is also involved in the stimulation of the profibrotic MAPK pathway. Together with molecules related to fibrosis, s(P)RR is also delivered to the cell surface by acidic organelles to be secreted into the peritoneal cavity, and may be useful as a biomarker for peritoneal injury and acidic organelle activity. This mechanism may serve as a new therapeutic target for peritoneal fibrosis.

As summarised in Fig. 7, we conclude that (P)RR and V-ATPase are responsible for the cellular fibrosis induced by TGF-β1 in HPMCs. We also propose that (P)RR and V-ATPase play an important role in acidic organelle function and are involved in the trafficking of profibrotic molecules such as FN1. Targeting this mechanism may lead to novel approaches to treat peritoneal fibrosis, which is a major complication in PD that limits PD duration.

## Methods

### Animals

All procedures were in accordance with the National Institutes of Health Guide for the Care and Use of Laboratory Animals, and were approved by the animal committee of Tohoku University (registration number: 2016MdA-092 and 2016MdA-170).

#### Animal study protocol 1: role of V-ATPase in MG induced peritoneal fibrosis in rats

We divided six to seven-week-old male Wistar rats (CLEA Japan, Tokyo, Japan) into 4 groups. Standard peritoneal dialysis fluid (PDF; Dianeal N PD-4, Baxter JAPAN, Tokyo, Japan) was injected peritoneally for 2 days, followed by peritoneal injection of PDF, PDF containing 100 nM BAF (Santa Cruz Biotechnology, Dallas, TX, USA), 20 mM MG (Sigma-Aldrich, St. Louis, MO, USA), or 100 nM BAF + 20 mM MG for another 7 days in the control (n = 7), BAF (n = 7), MG (n = 10), or MG + BAF (n = 6) groups, respectively. The total volume of peritoneally-injected solution was 20 mL. All rats were sacrificed at the end of the injection period under ketamine-xylazine anaesthesia. Parietal and visceral peritoneal samples were obtained and used for histological analysis including analysis of peritoneal thickness and immunohistochemical evaluation of TGF-β123, α-SMA, Fn1, Atp6v0c, and Atp6v1b1/2.

#### Animal study protocol 2: expression analysis of candidate molecules in MG-induced rat peritoneal mesothelial cell fibrosis

We divided six to seven-week-old male Wistar rats (SLC Japan, Shizuoka, Japan) into 3 groups. The control group (n = 10) received a single intraperitoneal injection of standard saline (Otsuka Pharmaceutical, Tokushima, Japan), whereas the MG group also received a single injection of saline containing either 1.0 mM (n = 12) or 20 mM (n = 11) MG. The volume of the intraperitoneal injection was 10 mL per 100 g body weight, which was performed under sevoflurane anaesthesia. On the 3rd day after injection, all animals were anesthetised. The omentum was digested 3 times with Hank’s balanced salt solution containing 0.125% trypsin and 240 μM ethylenediaminetetraacetic acid (EDTA) for 20 minutes at 37 °C, then cultured overnight. Subsequently, the RNA of these cells was extracted using RNeasy (Qiagen, Hilden, Germany) and mRNA expression of α-SMA, Mmp2, CK18, ZO-1, TGF-β1, (P)RR, Atp6v1b2, and Atp6v0c was evaluated by quantitative PCR.

#### Histological analysis of MG-induced peritoneal fibrosis in rats

Parietal and visceral specimens were fixed and stained with Masson trichrome for evaluation of peritoneal thickness. The thickening of submesothelial compact zone was defined that an area from the surface of the liver to peritoneal cavity in visceral peritoneum, an opposition parietal peritoneum, the thickening of the superficial layer which is light red cytoplasm surface of the green collagen was measured (Supplementary Fig. S6). The microscopic image was recorded at each of these ten positions of each animal. In addition, tissues were immunohistochemically stained with TGF-β123, α-SMA, Fn1, Atp6v0c, and Atp6v1b1/2 (Supplementary Table S1), and Histofine Simple Stain MAX PO (M) and (R) (Nichirei Bioscience, Tokyo, Japan) were used as secondary antibodies. Photographs were taken using a light microscope (DMi8; Leica, Wetzlar, Germany), and peritoneal membrane thickness was determined using imaging software (LAS X; Leica).

### Patients and sample collection

Data and, serum and urine samples were collected from 7 patients diagnosed with CKD on regular PD treatment between 2011 and 2016 at the Tohoku University Hospital. Sera were obtained from the peripheral blood were obtained, and stored at −80 °C until use. The study was performed under the declaration of Helsinki principles. The study protocol was approved by the Institutional Review Board of the Tohoku University School of Medicine (registration number: 2016-1-110). All participants provided written informed consent after a full explanation of the purpose of the study and the potential risk involved.

### Real-time quantitative PCR

Total RNA was extracted using RNeasy (Qiagen) and cDNA was synthesised using SuperScript III First-strand Synthesis SuperMix (Life Technologies, Carlsbad, CA, USA). Five nanograms of cDNA were used as a template for qPCR. The target sequence was amplified in duplicate with gene-specific primers (Supplementary Table S2) using SYBR Premix Ex Taq II reagent (TaKaRa, Shiga, Japan) and the CFX96 Real-Time PCR Detection System (Bio-Rad Laboratories, Hercules, CA, USA). Relative mRNA expression levels were normalised against glyceraldehyde 3-phosphate dehydrogenase (GAPDH) mRNA expression levels.

### Cell Culture

HPMCs were isolated from peritoneal dialysis effluents (PDEs) by using described methods^37,38^. The approximately 4.5 L of PDEs, obtained from each 7 stable patients with CKD on regular PD treatment (male:female = 5:2, Age: 66.7 ± 2.21 years old). Briefly, HPMCs were collected from PDEs impregnated with EDTA (2.5 mmol/L final concentration) by centrifugation for 10 min at 50 × g. The cell pellets were suspended in 1–3 mL of the culture M199 medium (Life Technologies), counted by cell counter, seeded in a 35-mm plastic dish, and incubated at 37 °C in 5% CO_2_. The collected cells were cultured in M199 medium supplemented with 10% fetal bovine serum (FBS; Life Technologies), 100 U/mL penicillin (Life Technologies), 100 µg/mL streptomycin (Life Technologies), 100 ng/mL hydrocortisone (Sigma-Aldrich), and insulin-transferrin-sodium selenite media supplement (Sigma-Aldrich). These cells were rinsed with 1 × phosphate-buffered saline (PBS) the next day and the culture medium was replaced every 2–3 days, thereby eliminating any contaminating blood components, for passage 1 or 2. The HPMC line, HMrSV5, was previously established^13^ and cultured in Dulbecco’s modified Eagle’s medium (Life Technologies) supplemented with 10% FBS, 100 U/mL penicillin, and 100 µg/mL streptomycin.

Prorenin and renin was obtained from Proteos (Kalamazoo, MI, USA), which was also used in our previous study^39^. We applied 10 µM losartan and 10 µM PD123319 (AT1 and AT2 receptor blocker, respectively; both Sigma-Aldrich) for 1 hour, prior to prorenin or renin stimulation. Human TGF-β1 (R&D Systems, Minneapolis, MN, USA) was applied for 24, 48, or 72 hours to the cultured medium. V-ATPase inhibitor BAF (Enzo Life Sciences, Farmingdale, NY, USA) or MEK inhibitor U0126 (Sigma-Aldrich) was applied to the cultured medium 1 hour prior to the TGF-β1 stimulation.

### Immunohistochemistry

HPMCs and HMrSV5 cells were seeded and cultured overnight, fixed with 4% paraformaldehyde for 15 minutes, and permeabilised with 0.1% Triton-X100 (Sigma-Aldrich) in 1 × phosphate-buffered saline (PBS) for 10 minutes. After treatment with 3% normal goat serum (NGS; Sigma-Aldrich) in PBS, cells were incubated with primary antibodies (Supplementary Table S1) overnight at 4 °C. Slides were incubated with anti-mouse-FITC (Millipore, Darmstadt, Germany) and anti-rabbit-Alexa 555 or 488 (Life Technologies), and mounted onto glass slides with DAPI Fluoromount-G (SouthernBiotech, Birmingham, AL, USA). Fluorescence was observed using a Nikon C2i confocal microscope (Nikon Instech, Tokyo, Japan). Antibody specificity toward (P)RR was determined previously^40^.

### Imaging of endosomes/exosomes

HPMCs and HMrSV5 cells were loaded with 50 nM LysoTracker Red DND-99 (Life Technologies) in culture medium for 1 hour at 37 °C. After incubation, the medium was changed, and the cells were mounted with DAPI Fluoromount-G, and scanned using a Nikon C2i confocal microscope.

### Subcellular fractions

HMrSV5 cells were crudely separated into subcellular fractions (cytosol, membranous organelle, nuclear membrane, and insoluble protein) using the EzSubcell Extract kit (WSE-7421, ATTO, Tokyo, Japan) (Fig. 2C). For enrichment, the membranous organelle fraction was suspended in 3 mL of 19% (w/v) OptiPrep (Sigma-Aldrich) for a step-gradient containing 1.5 mL of 30%, 1.5 mL of 26%, 2.0 mL of 22%, 3.0 mL of 19% (sample), 2.0 mL of 14%, 1.0 mL of 10%, and 1.0 mL of 6% OptiPrep each. Each density of OptiPrep was prepared by diluting 50% OptiPrep in a buffer containing 10 mM Tris-HCl, pH 7.4, 250 mM sucrose, and 1.0 mM EDTA. Ultracentrifugation was performed at 150,000 × *g* for 16 hours. The 13 subcellular fractions were collected from the bottom of the tube. Each fraction was diluted with B88 buffer (20 mM HEPES-KOH, pH 7.4, 250 mM sorbitol, 150 mM potassium acetate, and 5.0 mM magnesium acetate) plus protease inhibitors (Roche Indianapolis, IN, USA), phosphatase inhibitors (Sigma-Aldrich), and 0.3 mM dithiothreitol (Wako, Osaka, Japan), and membranes were collected by centrifugation at 150,000 × *g* for 30 minutes. All ultracentrifugation steps utilised a swing-out rotor (P40ST, Hitachi, Tokyo, Japan).

### Short interfering RNA (siRNA) transfection

A short interfering RNA (siRNA) for (P)RR (LQ-013647–01) and a control scrambled siRNA (scRNA, D-001810–10) were purchased from Dharmacon (GE Healthcare, Buckinghamshire, UK). HMrSV5 cells were seeded at a density of 2 × 10^5^ cells/well in 12-well plates and transfected with 10 nM of either siRNA or scRNA using Lipofectamine 2000 (Life Technologies). Transfected cells were used for experiments 48 hours after transfection.

### Western blot analysis

HMrSV5 cells were incubated in serum-free medium for 24 hours, then stimulated with renin or prorenin at the indicated times and concentrations. For TGF-β1 stimulation, 0.2% FBS medium was used instead of serum-free medium for synchronisation of the cell cycle and maintenance of cellular viability during TGF-β1 stimulation, according to a previous study^30^. Subcellular organelle fractions were separated using EzSubcell Extract (WSE-7421, ATTO) following manufacturer protocol. Whole cell protein was extracted with RIPA buffer (Cell Signaling Technology, Danvers, MA, USA) containing 1.0 mM phenylmethylsulphonyl fluoride (Thermo Fischer Scientific, Waltham, MA, USA) and protease inhibitor (Roche, Basel, Switzerland). Following protein separation by denaturing gel electrophoresis, the membranes were incubated with primary antibodies (Supplementary Table S1) overnight at 4 °C. Membranes were incubated with horseradish-peroxidase-conjugated anti-rabbit IgG, anti-mouse IgG, or anti-goat IgG (all Santa Cruz) secondary antibodies, or Alexa Fluor 488-labelled anti-mouse IgG secondary antibody (Life Technologies) for 1 hour. The membranes were visualised using an ECL western blotting detection system (Thermo Fischer Scientific) using VersaDocMP5000 and ChemiDoc MP (Bio-Rad). The relative expression level of each protein was normalised against β-actin (Santa Cruz).

### Biochemical assays

Prorenin and s(P)RR concentration was measured using a Human Prorenin ELISA Kit (Innovative Research, Novi, MI, USA) and a soluble (Pro)renin Receptor Assay Kit (IBL, Fujioka, Japan), respectively. Albumin, α_1_-macroglobulin, α_2_-microglobulin, IgG, and renin concentrations were measured using a modified bromocresol purple method, turbidimetric immunoassay, latex agglutination turbidimetry, and immunoradiometric assay, respectively.

### Statistical analysis

Continuous values are given as the means ± SE. Statistical comparisons were made using an unpaired *t*-test for two-group comparison, and analysis of variance (ANOVA) followed by a Tukey test as the post-hoc test to determine multiple comparisons of differences among the groups. P-values of P *< *0.05 were considered to be statistically significant.

## Electronic supplementary material

Supplementary Figures and Tables
