# Supplementary material for: Acidic organelles mediate TGF-β1-induced cellular fibrosis via (pro)renin receptor and vacuolar ATPase trafficking in human peritoneal mesothelial cells
Source: Sci Rep. 2018 Feb 8;8:2648. doi: 10.1038/s41598-018-20940-x (PMC5805675; doi:10.1038/s41598-018-20940-x)
Supplement: Supplementary file 1 — Supplementary Figures and Tables [file 41598_2018_20940_MOESM1_ESM.pdf]

SUPPLEMENTARY INFORMATION

Acidic organelles mediate TGF-β1-induced cellular fibrosis via (pro)renin receptor and vacuolar ATPase trafficking in human peritoneal mesothelial cells

Ikuko Oba-Yabana[1,2], Takefumi Mori[1,2,*], Chika Takahashi[3], Takuo Hirose[1,2,4], Yusuke Ohsaki[3], Satoshi Kinugasa[2], Yoshikazu Muroya[2], Emiko Sato[1], Geneviève Nguyen[4], Rémi Piedagnel[5.6], Pierre M Ronco[5,6,7], Kazuhito Totsune[8] & Sadayoshi Ito[1]

[1]Division of Nephrology, Endocrinology and Vascular Medicine, Tohoku University Graduate School of Medicine, Sendai, Japan.

[2]Division of Nephrology and Endocrinology, Tohoku Medical and Pharmaceutical University, Sendai, Japan.

[3]Division of Integrated Renal Replacement Therapy, Tohoku University Graduate School of Medicine, Sendai, Japan.

[4]Center for Interdisciplinary Research in Biology, Collège de France, Paris, France.

[5]INSERM, UMR_S 1155, F-75020, Paris, France.

[6]Sorbonne Universités, UPMC Univ Paris 06, UMR_1155, F-75005, Paris, France.

[7]Department of Nephrology, AP-HP, Hôpital Tenon, Paris, France.

[8]Department of Social Welfare, Faculty of Synthetic Welfare, Tohoku Fukushi University, Sendai, Japan.

*Correspondence and requests for materials

Takefumi Mori, MD, PhD,

Division of Nephrology and Endocrinology, Tohoku Medical and Pharmaceutical University, 1-12-1, Fukumuro, Miyagino, 983-8512, Sendai, Japan.

Tel: +81–22–259–1221, Fax: +81–22–259–1232

E-mail: tmori@hosp.tohoku-mpu.ac.jp

Supplemental Figure S1

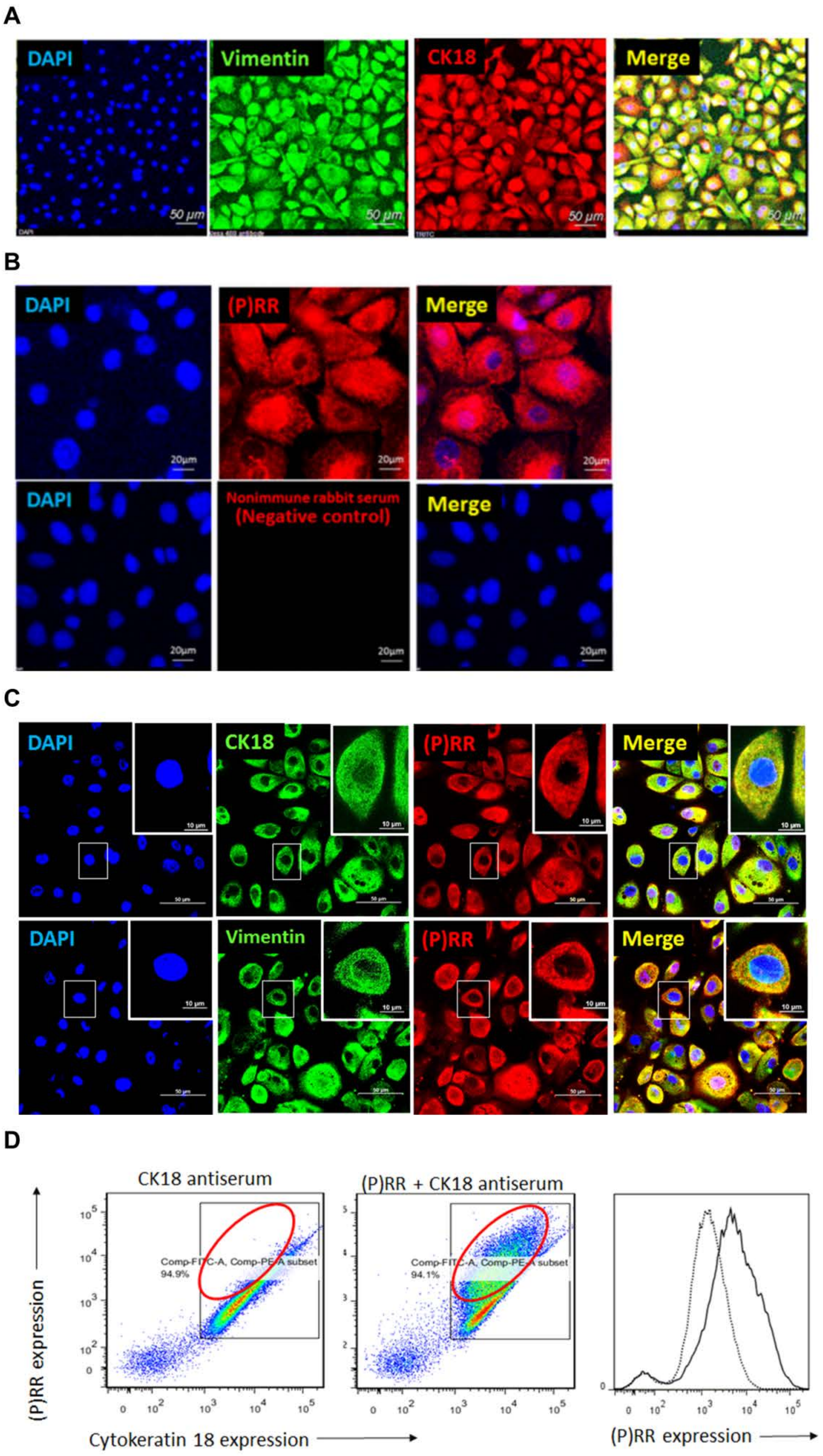

Supplementary Figure S1. Expression of the (pro)renin receptor determined in human peritoneal mesothelial cell (HPMC) culture. HPMCs were isolated from peritoneal dialysis effluents (PDEs), obtained from stable patients with CKD on regular PD treatment. Cultured HPMCs were incubated with anti-(P)RR antibody (1:1000, in-house(34)) or cytokeratin 18 antibody (1:50, a marker for HPMCs, DAKO), followed by a secondary anti-rabbit antibody-PE and anti-mouse antibody-FITC (both 1:5000, Imgenex, San Diego, CA, USA). Labelled cells were analysed using a FACS Canto II (Becton Dickinson, Franklin Lakes, NJ, USA), and data were analysed using FlowJo software (Tree Star, Ashland, OR, USA). (**A**) Immunohistochemistry of HPMCs obtained from PDEs to verify the specificity of the cells. All of the cells were stained with anti-cytokeratin 18 (CK18) and anti-vimentin for HPMC markers. Scale bars, 50 μm. (**B**) Immunohistochemistry of the pro(renin) receptor [(P)RR] in HPMCs. Specificity of the antibody to (P)RR was verified. HPMCs were stained with anti-(P)RR with non-immune rabbit serum as a negative control. Scale bars, 20 μm. (**C**) Immunohistochemistry to determine expression of (P)RR in HPMCs obtained from PDEs. (P)RR immunostaining was observed in cells stained with CK18 and vimentin. Scale bars, 50 μm. Magnified views of the white boxes are shown in the upper right corner. Scale bars, 10 μm. (**D**) Flow cytometric analysis of (P)RR expression in cells from PDEs to determine whether (P)RR is expressed in CK18-positive HPMCs. Representative flow cytogram of cultured HPMCs labelled with anti-CK18 antibody and non-immune serum (left), or anti-CK18 antibody and (P)RR antiserum (middle). Approximately 95% cells were CK18-positive in cultured HPMCs. Cells in red circles were detected as (P)RR-positive. Right: representative histograms of (P)RR expression in HPMCs. Cells treated with (P)RR antiserum are indicated with a solid line, and cells treated with non-immune serum are shown using a dotted line.

Supplemental Figure S2

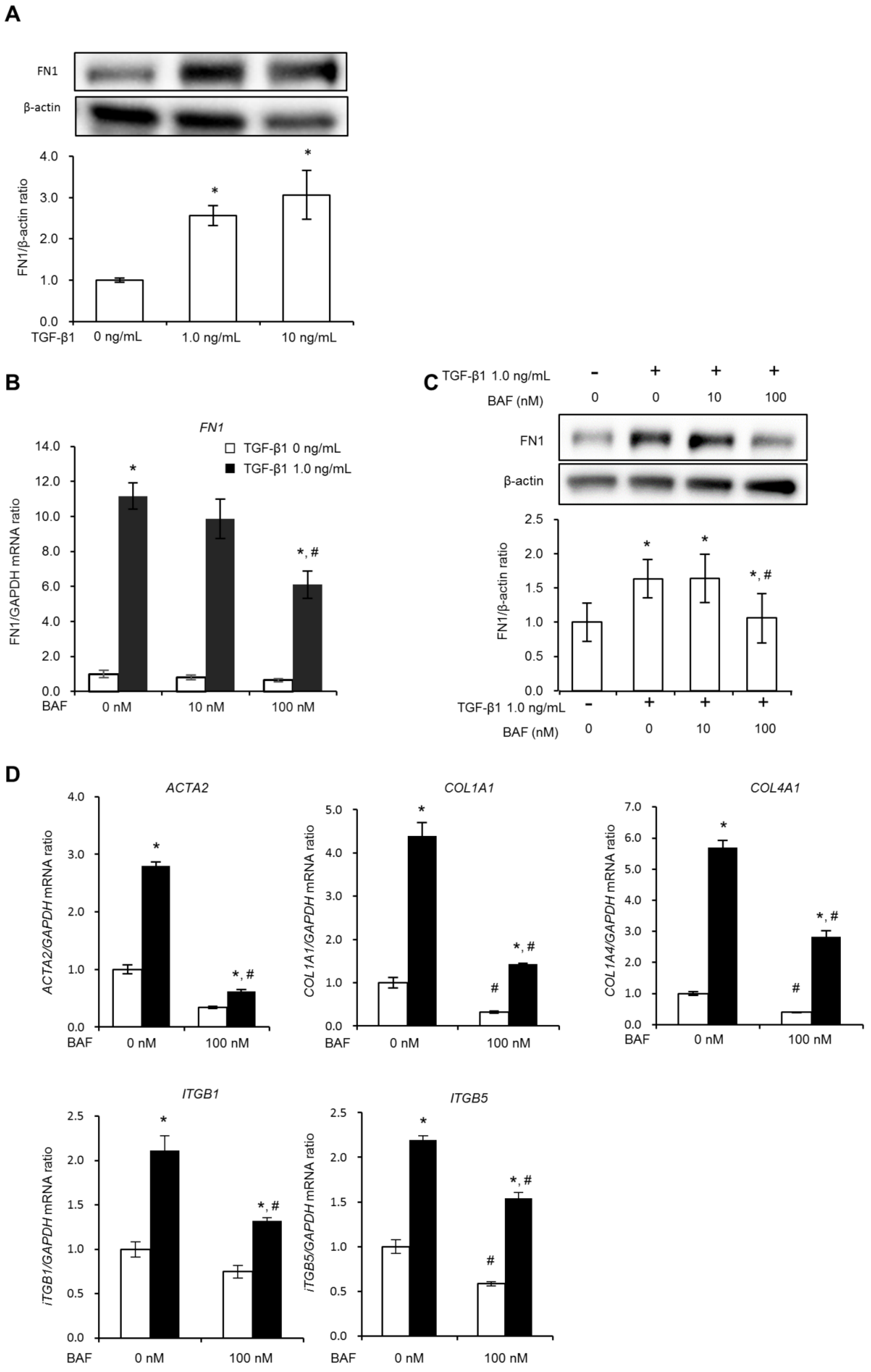

Supplementary Figure S2. BAF inhibit TGF-β1-induced fibrosis in cultured HPMC (HMrSV5 cells). (A) TGF-β1 stimulation increases FN1 expression in HMrSV5 cells. HMrSV5 cells were stimulated with 0, 1, or 10 ng/mL TGF-β1 for 24 hours. Values are expressed as the means ± SE (n = 4). *, P < 0.05 vs 0 ng/mL TGF-β1. (**B**) Dose response of the V-ATPase inhibitor BAF on the expression of molecules related to TGF-β1-induced fibrosis in HMrSV5 cells. *, P < 0.05 vs 0 ng/mL TGF-β1; #, P < 0.05 vs 0 nM BAF. (**C**) Western blot analysis of FN1 expression. Values are expressed as the means ± SE (n = 4). *, P < 0.05 vs 0 ng/mL TGF-β1; #, P < 0.05 vs 0 nM BAF. (**D**) mRNA expression levels of fibronectin (*FN1*), α-SMA (*ACTA2*), collagen I (*COL1A1*), collagen IV (*COL4A1*), integrin 1 (*ITGB1*), and 5(*ITGB5*) were normalised with *GAPDH* mRNA. Values are expressed as the means ± SE (n = 3). *, P < 0.05 vs 0 ng/mL TGF-β1; #, P < 0.05 vs 0 nM BAF.

Supplemental Figure S3

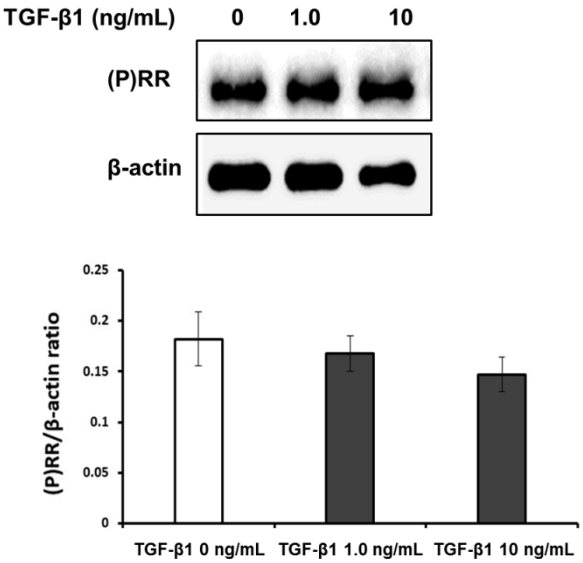

Supplementary Figure S3. Effect of TGF-β1 on (P)RR expression in cultured HPMC (HMrSV5 cells). HMrSV5 cells were treated with 0, 1 or 10 ng/mL TGF-β1 for 24 h. (P)RR protein levels were evaluated by western blot analysis. TGF-β1 treatment had no significant effect on (P)RR expression. Values of four experiments for each group are expressed as mean ± SE ($n = 4$, $P = 0.511$).

Supplemental Figure S4

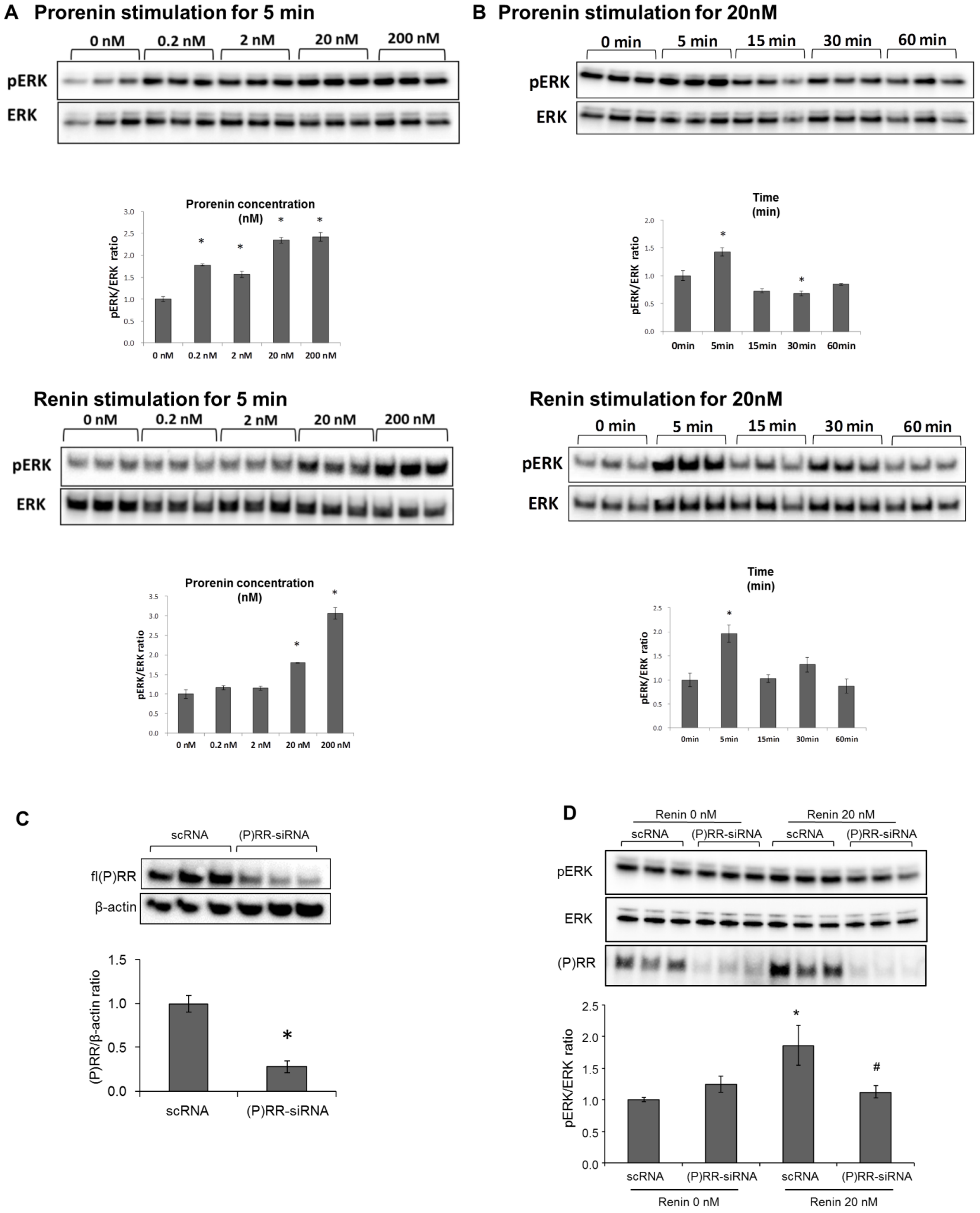

Supplementary Figure S4. Effect of (P)RR on ERK1/2 phosphorylation in cultured HPMC (HMrSV5 cells). (**A**) Dose response of prorenin (upper) or renin (bottom) stimulation on ERK1/2 phosphorylation. HMrSV5 cells were treated with prorenin or renin for 5 minutes, and then ERK1/2 phosphorylation was analysed by western blotting. Values of three experiments per concentration are expressed as the means ± SE (n = 3). *Significant difference compared with 0 nM (P < 0.05). (**B**) Time course of prorenin/renin stimulation on ERK1/2 phosphorylation. HMrSV5 cells were treated with 20 nM prorenin (upper) or 20 nM renin (bottom), and ERK1/2 phosphorylation was analysed by western blotting. Values of three experiments per time course are expressed as the means ± SE (n = 3). *Significant difference compared with 0 minutes (P < 0.05). (**C**) (P)RR knockdown by (P)RR-siRNA to determine the specific role of (P)RR on ERK1/2 phosphorylation. HMrSV5 cells were transfected with (P)RR-siRNA for 48 hours and (P)RR protein levels were evaluated by western blot analysis. ScRNA was used as a negative control. Values of three experiments for each group are expressed as the means ± SE (n = 3). *Significant difference compared with scRNA. (P < 0.05). (**D**) Effect of (P)RR knockdown on renin-stimulated ERK1/2 phosphorylation. HMrSV5 cells were treated with scRNA or (P)RR-siRNA for 48 hours and stimulated with 20 nM renin (n = 6) for 5 minutes. Prior to prorenin/renin stimulation, cells were pretreated with 10 μM losartan (an AT1 receptor blocker) and 10 μM PD123319 (an AT2 receptor blocker) for 1 hour. Value of six experiments for each group are expressed as the means ± SE (n = 6). *Significant difference compared with 0 nM renin treatment; #significant difference compared with scRNA 20 nM renin treatment (P < 0.05).

Supplemental Figure S5

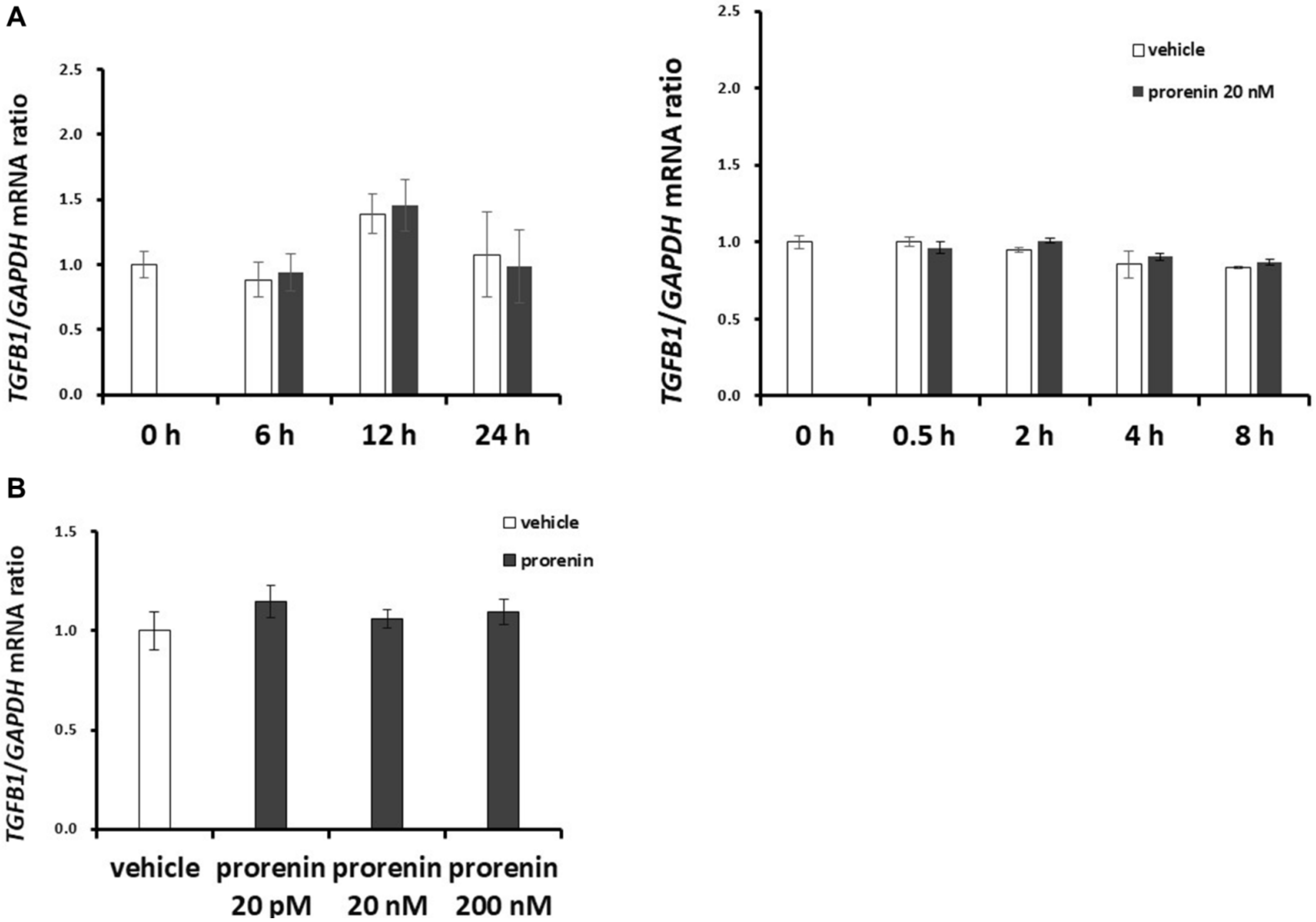

Supplementary Figure S5. Effect of prorenin on TGF-β1 expression in cultured HPMC (HMrSV5 cells). (**A**) Time course of prorenin stimulation on TGF-β1 (*TGFB1*) mRNA expression in the presence of AT1 receptor blocker (10 μM losartan) and AT2 receptor blocker (10 μM PD123319) (Left; means ± SE, n = 3, P = 0.948), and in the absence of losartan and PD123319 treatment (Right; means ± SE, n = 3, P = 1.000). HMrSV5 cells were stimulated with 20 nM prorenin for the indicated times, and mRNA levels were evaluated by real-time quantitative PCR. No significant difference was observed. (**B**) Dose response of prorenin treatment on TGF-β1 (*TGFB1*) mRNA expression in the absence of losartan and PD123319. HMrSV5 cells were stimulated with indicated concentrations of prorenin for 4 h, and mRNA levels were evaluated by qPCR. Values of three experiments for each group are expressed as means ± SE (n = 3, P = 0.550). No significant difference was observed.

Supplemental Figure S6

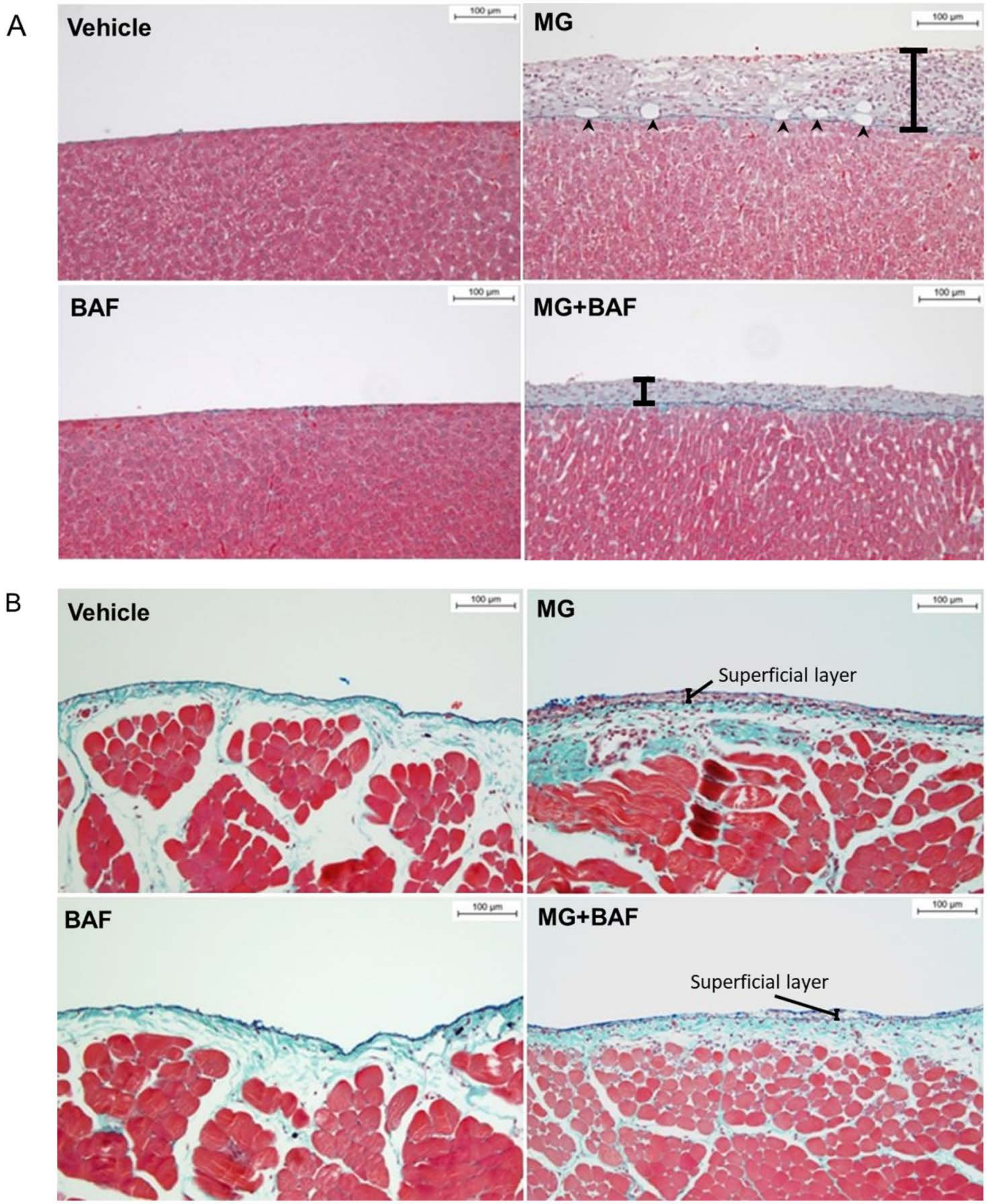

Supplementary Figure S6. Histological analysis of peritoneal fibrotic area. (**A**) The thickness of visceral peritoneum was measured from the surface of the liver to peritoneal cavity (Bars). (**B**) The thickness of parietal peritoneum was measured the superficial layer which is light red cytoplasm surface (Bars).

Supplementary Table S1 : List of antibodies used.

Antigen	Company	Catalog number	Host	IHC	IF	FACS	WB
ACTA (α-SMA)	Dako	M0851	mouse	1:200			
ACTB (β-actin)	Santa Cruz	sc-47778	mouse				1:5000
ATP1A1 (Na^+K^+ATPase)	Santa Cruz	sc-21712	mouse				1:200
ATP6AP2 [(P)RR]	In house (ref. 40)		rabbit		1:1000	1:1000	1:1000
ATP6V0c	Abcam	ab104374	rabbit	1:100			1:1000
ATP6V1B1/2	Santa Cruz	sc-55544	mouse	1:800	1:100		1:100
ATP6V1C1	Santa Cruz	sc-21211	goat				1:200
COL3A1	Novus biologicals	NB600-594	rabbit				1:1000
COL4	Abcam	ab6586	rabbit		1:200		
EDA-FN1	Sigma-Aldrich	F6140	mouse		1:100		1:400
EEA-1	Abcam	ab70521	mouse		1:1000		
EEA-1	Cell signaling	2411	rabbit				1:1000
ERK	Cell signaling	4377	rabbit				1:1000
FN1	Sigma-Aldrich	F3648	rabbit	1:400	1:100		1:1000
GM130	ECM Biosciences	GM3421	mouse		1:50		
GM130	Cell signaling	12480	rabbit				1:1000
Histon H4	Cell signaling	2592	rabbit				1:1000
KRT18 (CK18)	Abcam	ab93741	rabbit		1:500		
KRT18 (CK18)	Dako	M7010	mouse			1:50	
LAMP1	Abcam	ab25630	mouse		1:100		
LAMP2	Sigma-Aldrich	L0668	rabbit				1:1000
PDI	Abcam	ab2792	mouse		1:100		1:1000
pERK	Cell signaling	4696	mouse				1:1000
pSMAD2/3	Cell signaling	8828	rabbit				1:1000
SMAD2/3	Cell signaling	8685	rabbit				1:1000
TGFB123	Santa Cruz	sc-7892	rabbit	1:50			
TUBA (α-tubulin)	Cell signaling	2144	rabbit				1:1000
VIM	Dako	M0725	mouse		1:100		

Supplementary Table S2 : Primer sequences for RT-PCR.

Gene Name	Forward primer	Reverse primer
(human)		
GAPDH	5′-GCACCGTCAAGGCTGAGAAC-3′	5′-TGGTGAAGACGCCAGTGGA-3′
β-actin	5′- TGGCACCCAGCACAATGAA-3′	5′- CTAAGTCATAGTCCGCCTAGAAGCA-3′
ATP6AP2 [(P)RR]	5′-CCTCCCTCATTAGGAAGACAAGGAC-3′	5′-TCGAATCTTCTGGTTTGTCATCCT-3′
FN1	5′-GCAGTGGCTGAAGACACAAGG-3′	5′-TGTAGGTGAATGGTAAGACACATGG-3′
ACTA2	5′-ATAGAACATGGCATCATCACCAAC-3′	5′-GGGCAACACGAAGCTCATTGTA-3′
COL1A1	5′- GCTTGGTCCACTTGCTTGAAGA-3′	5′- GAGCATTGCCTTTGATTGCTG-3′
COL4A1	5′- CAGCCGCTGCCAAGTCTGTA-3′	5′- AGGTCAATGAAGCAGGGTGTGTTAG-3′
ITGB1	5′- TCCCTAAGTCAGCAGTAGGAACATT-3′	5′- CCCGTTCTTGCAGTAAGATTTGTA-3′
ITGB5	5′- AGGTGATCACATGGGTGGACA-3′	5′- GAACATCATGACGCAGTCCTTG-3′
SMAD2	5′- GCTCTGGCGTCTACTGCATTTC-3′	5′- TGCCATTGGCTGATGCATTTA-3′
TGFBR2	5′- TGCAAAGGTTTGGAAATAGAACCTC-3′	5′- CATGCCCTACGGTGCAAGTG-3′
REN	5′- GTGGAGCCAACCCATGAAGA-3′	5′- TTGGACGAACCAGTGTCAAAGA-3′
(rat)		
Gapdh	5′-GGCACAGTCAAGGCTGAGAATG-3′	5′- ATGGTGGTGAAGACGCCAGTA-3′
Tgfb1	5′- TGCGCCTGCAGAGATTCAAG-3′	5′- AGGTAACGCCAGGAATTGTTGCTA-3′
Acta2 (α-SMA)	5′-AGCCAGTCGCCATCAGGAAC-3′	5′- GGGAGCATCATCACCAGCAA-3′
Mmp2	5′-GGACAGTGACACCACGTGACAA-3′	5′-TTTCCAAAGTGCTGGCAGAATAGAC-3′
Krt18 (CK18)	5′- CGTCTTGCCGCTGATGACTTTA-3′	5′-GAGCGCTTCGATTTCTGTCTCC-3′
Zo-1	5′- CGGAAATGTGTAAATCACCTGGAA-3′	5′- CATGCGTCCTGAACACATCAAAC-3′
Atp6ap2 [(P)RR]	5′-AGGACCATCCTTGAGACGAAACA-3′	5′-GGCCAAGCCAGTCATAATCCAC-3′
Atp6v0c	5′- GCAGACATGGCTGACATCAAGAA-3′	5′- CCATAACCACTGGGATGATGGAC-3′
Atp6v1b2	5′- GAAGCCCTGACCTCAGATGACC -3′	5′- ACTGCCAGCCAATGTCCAAAG-3′
